# Functional Enterospheres Derived *In Vitro* from Human Pluripotent Stem Cells

**DOI:** 10.1016/j.stemcr.2017.07.024

**Published:** 2017-08-31

**Authors:** Rohan R. Nadkarni, Soumeya Abed, Brian J. Cox, Sonam Bhatia, Jennifer T. Lau, Michael G. Surette, Jonathan S. Draper

**Affiliations:** 1 McMaster Stem Cell and Cancer Research Institute, Michael G. DeGroote School of Medicine, McMaster University, Hamilton, ON L8N 3Z5, Canada; 2 Department of Pathology and Molecular Medicine, McMaster University, Hamilton, ON L8N 3Z5, Canada; 3 Department of Biochemistry and Biomedical Sciences, McMaster University, Hamilton, ON L8N 3Z5, Canada; 4 Department of Physiology, University of Toronto, Toronto ON, Canada; 5 Michael G. DeGroote Institute for Infectious Disease Research, McMaster University, Hamilton, ON L8N 3Z5, Canada; 6 Farncombe Family Digestive Health Research Institute, McMaster University, Hamilton, ON L8N 3Z5, Canada; 7 Department of Medicine, McMaster University, Hamilton, ON L8N 3Z5, Canada; 8 Department of Obstetrics and Gynaecology, University of Toronto, Toronto ON, Canada

**Keywords:** enterospheres, intestinal organoids, intestine, human pluripotent stem cells, *in vitro* differentiation, Wnt signaling, innate immunity

## Abstract

Intestinal organoids derived from human pluripotent stem cells (hPSCs) are valuable *in vitro* research models that enable simplified access to human gastrointestinal tissues. Here, we report the *in vitro* generation of enterospheres (hEnS) from hPSC-derived gastrointestinal epithelial precursors. hEnS are cystic spheroids with a simple uniform structure composed entirely of intestinal epithelium. hEnS express markers of mature brush border cells and share a transcriptome profile similar to that of more mature intestinal organoids. Modulation of signaling cues enables control of hEnS growth and differentiation, including long-term propagation. We show that hEnS can be exploited for functional studies: hEnS display an innate immune response when treated with enteric pathogens, and transgenic modification of hEnS with a fluorescence cell-cycle reporter enables hEnS-forming stem cell enrichment. Our work establishes hEnS as an accessible and tractable *in vitro* modeling system for studying human gastrointestinal biology.

## Highlights

• Generated entirely *in vitro*, hEnS exhibit features of intestinal epithelium
• Long-term growth and differentiation of hEnS requires specific media conditions
• hEnS elicit an innate immune response after treatment with enteric pathogens
• hEnS can be transgenically modified to enrich for stem cells

## Teaser

Organoids derived from human pluripotent stem cells (hPSCs) represent an attractive system for investigating human development and disease *in vitro*. Nadkarni et al. show that hPSC-derived cystic intestinal organoids, or enterospheres, have a uniform epithelial structure, are easily accessible to genetic modification, and contain functional intestinal cell types that provide an *in vitro* modeling system for studying human gastrointestinal biology.

## Introduction

Human pluripotent stem cells (hPSCs) can differentiate into all specialized cell types of the body, providing material suitable for a range of applications, including regenerative medicine. hPSCs facilitate research questions that are impractical or difficult using tissue samples derived from patients, including the study of developmental lineage specification. Differentiation studies using hPSCs have helped uncover novel information about human development, such as the roles of signaling pathways in lineage commitment, and have provided evidence of intermediate cell populations during differentiation (Murry and Keller, 2008, Zhu and Huangfu, 2013, Zorn and Wells, 2009).

The differentiation of hPSCs into functional cell types has been enhanced by the production of organoids, 3D structures mimicking the structural and functional properties of *in vivo* organs (Lancaster and Knoblich, 2014). Organoids can be derived from primary adult stem cells as well as hPSC sources (Fatehullah et al., 2016, Huch and Koo, 2015, Nadkarni et al., 2015), but hPSC-derived organoids offer some important advantages, including increased accessibility and an unlimited supply of the starting material. Therefore, the recent establishment of intestinal organoid cultures from hPSCs represents a major advance toward creating a model system of the human intestine (Fordham et al., 2013, Forster et al., 2014, Spence et al., 2011). Organoids generated using various methods have been shown to contain cell types with properties of intestinal stem and differentiated epithelial cells, as well as stromal components. Most studies have relied upon *in vivo* engraftment either to achieve maturation of organoids (Finkbeiner et al., 2015, Spence et al., 2011, Watson et al., 2014) or to derive the organoid precursor cells themselves (Forster et al., 2014). Although these reports have been highly instructive, a completely *in vitro* approach for the *de novo* production of uniform intestinal organoids containing differentiated cell types would advance gastrointestinal (GI) research.

Here, we report the *in vitro* generation from hPSCs of enterospheres (hEnS) with intestinal cell maturation features akin to those previously obtained via *in vivo* engraftment. hEnS express markers of intestinal epithelial cells types and are similar in gene expression to primary human intestine. We provide detailed insights into the properties of hEnS, and show that they respond to signaling cues during growth and differentiation comparably with primary and hPSC-derived intestinal organoids generated by other methods (Fordham et al., 2013, Forster et al., 2014, Sato et al., 2011). In doing so, we establish hEnS as a research tool that will have utility for the broader stem cell and gastrointestinal research communities.

## Results

### Spheroid Production from hESC-Derived Endoderm Tissues

**Figure 1:**
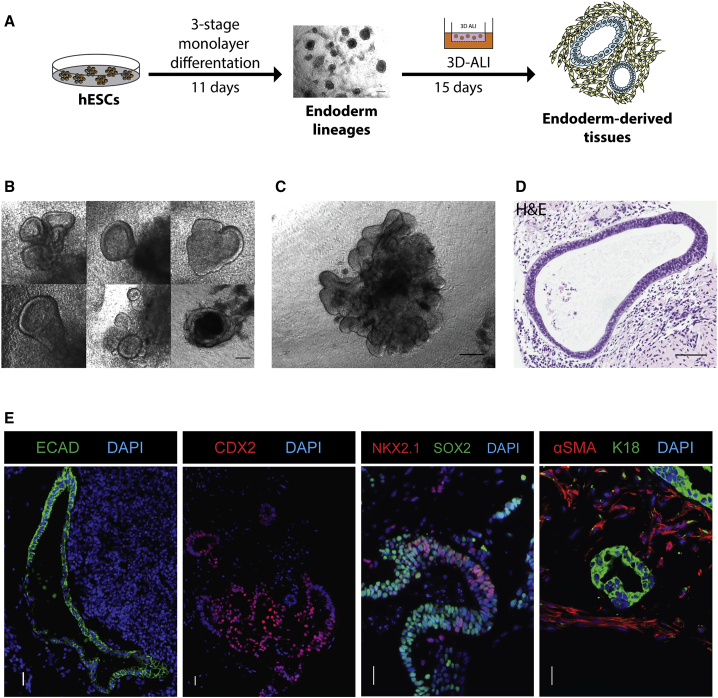
Generation of Endoderm-Derived Tissues from hESCs *In Vitro* (A) Schematic of *in vitro* stepwise differentiation scheme into endoderm-derived tissues. (B) Emergence of budding structures in the stage-4 3D conditions at day 7. (C) Budding structures develop into complex organoids by day 10. Images in (B) and (C) are of structures made from H1 hESCs. (D) H&E staining of 3D tissues showed organized tubular epithelia surrounded by cells with mesenchymal properties. Image shown is of H9 hESC-derived tissue. (E) Immunofluorescence staining of H1 and H9 hESC-derived 3D tissues shows that tubular structures express epithelial markers ECAD and K18, while subsets of surrounding cells express mesenchymal marker α-SMA. Expression of CDX2, an intestinal epithelial marker, and early lung epithelial markers NKX2.1 and SOX2 are evident. Scale bars, 200 μm (A), 50 μm (B and E), 250 μm (C), and 100 μm (D). See also Figure S1 and Movie S1.

Since *in vitro* culture duration for the differentiation of other cell types from hPSCs appears to correlate with maturation status (Lundy et al., 2013, Nicholas et al., 2013, Yang et al., 2014, Zhang et al., 2009), we tested whether the combination of extended *in vitro* differentiation with an unbiased method for isolating the gut-tube progenitor populations would enhance the maturation status of hPSC-derived tissues compared with current *in vitro* methods. To obtain tissue to evaluate this strategy, we employed a multistage 11-day monolayer differentiation protocol (D'Amour et al., 2005, Green et al., 2011) that produced a mixture of posterior (CDX2-expressing mid/hindgut cells) and anterior (NKX2-1-expressing putative lung progenitors) endoderm-derived lineages (Figure S1). This population was then used to initiate the production of 3D tissues: the day-11 monolayer cultures were mechanically dissociated and seeded into a Matrigel-based 3D growth environment (Figures 1A and S2). Small cellular aggregates obtained via mechanical dissociation of the monolayer cultures gave rise to multiple organized epithelial “buds” (Figures 1B and S2B) that migrated out of the cell aggregates (Movie S1) during the first 4 days of culture; the transfer of singularized cells (seeded at 50,000 or 70,000 per well) from the day-11 monolayer into 3D cultures did not facilitate appreciable bud formation (Figure S2A). By day 15 of 3D culturing, the buds had extensively self-organized to form complex structures (Figure 1C). These structures comprised E-cadherin (ECAD)- and cytokeratin 18 (K18)-expressing epithelial tubules surrounded by mesenchymal tissues that stained positive for α-smooth muscle actin (α-SMA) (Figures 1D and 1E). The mesenchymal component appeared to be necessary for epithelial integrity and growth, as epithelial tubules isolated by microdissection degenerated when not co-cultured with mesenchymal cells isolated from the 3D structures (Figure S2C). Analysis of protein expression within the epithelial tubules revealed the presence of CDX2^+^ cells (Figure 1E), as well as cells expressing markers consistent with an early lung bud fate, including the transcription factors NKX2.1, SOX2, and p63 (Figure 1E). Therefore, the 3D culture environment generated complex 3D structures with organized epithelial tubules containing gastrointestinal and lung cell populations resembling those of the developing fetal gut tube.

**Figure 2:**
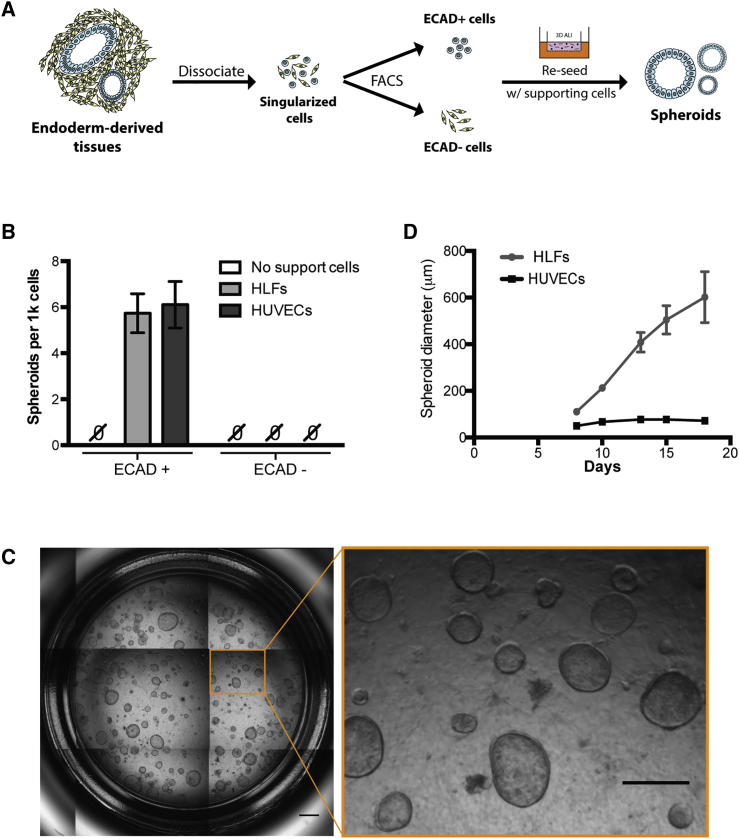
Generation of Spheroids from Purified Epithelial Progenitors in Endoderm-Derived Tissues (A) Schematic of spheroids generation *in vitro*. (B) Number of spheroids produced from ECAD^+^ and ECAD^−^ cells with or without supporting cell types HLFs and HUVECs (mean ± SEM, n = 8 and n = 2 independent experiments for HLFs and HUVECs, respectively). (C) Whole-well scan of day-18 spheroids derived from ECAD^+^ cells with HLFs in MTEC medium. Scale bar, 1 mm; scale bar in inset, 500 μm. (D) Comparison of spheroid diameter growth between HLF- and HUVEC-assisted spheroids (mean ± SEM, n ≥ 12 individual spheroids tracked). All data shown are for spheroids derived from H1 hESCs. See also Figure S2.

We next evaluated fluorescence-activated cell sorting (FACS)-based isolation as a strategy for purifying progenitor populations, using clonal organoid formation and long-term propagation as a stringent assay for the presence of self-renewing progenitor populations. We opted to perform an unbiased isolation of epithelial cells from dissociated day-15 3D tissues by performing FACS using the pan-epithelium marker ECAD (Figures 2A and S2D). We cultured the sorted cells in the same 3D Matrigel-based growth environment supplemented with MTEC medium containing components compatible with the growth of a range of cell types (Baten et al., 1992, Jumarie and Malo, 1991, Takenaka et al., 2014). No structures formed within 2 weeks post plating for either ECAD^+^ or ECAD^−^ cells obtained from the 3D endoderm-enriched tissues (Figure 2B). However, when sorted ECAD^+^ cells were co-cultured with human lung fibroblasts (HLFs) or human umbilical vein endothelial cells (HUVECs) at a ratio of 1:1, cystic organoids (herein termed spheroids) were observed within 6 days at a frequency of ∼1 per 200 seeded ECAD^+^ cells from H1 hESCs (Figures 2B and 2C). The spheroid-forming frequency of ECAD^+^ cells derived from H9 hESCs was ∼1:2,500 and ∼1:2,000 cells when co-cultured with HLFs and HUVECs, respectively (data not shown). These spheroids had a uniform epithelial structure, and the morphology was consistent across spheroid units (Figure 2C). Examination of spheroid growth kinetics showed that when co-cultured with HLFs, spheroids were maintained and grew in size over the course of the following 2 weeks (Figure 2D). In contrast, spheroids that formed with the assistance of HUVECs did not grow in size and cultures collapsed after 2 weeks (Figure 2D), so subsequent experiments utilized the HLF co-culture method. No structures were observed in ECAD^−^cultures with or without supporting cell types despite prolonged culture over 2 weeks (Figure 2B). These data show that FACS-based purification of ECAD^+^ cells isolated spheroid precursor cells from the 3D endoderm-derived tissues capable of forming spheroids when co-cultured with HLFs.

### Molecular Characterization of Spheroids Reveals Intestinal Lineage Enrichment

**Figure 3:**
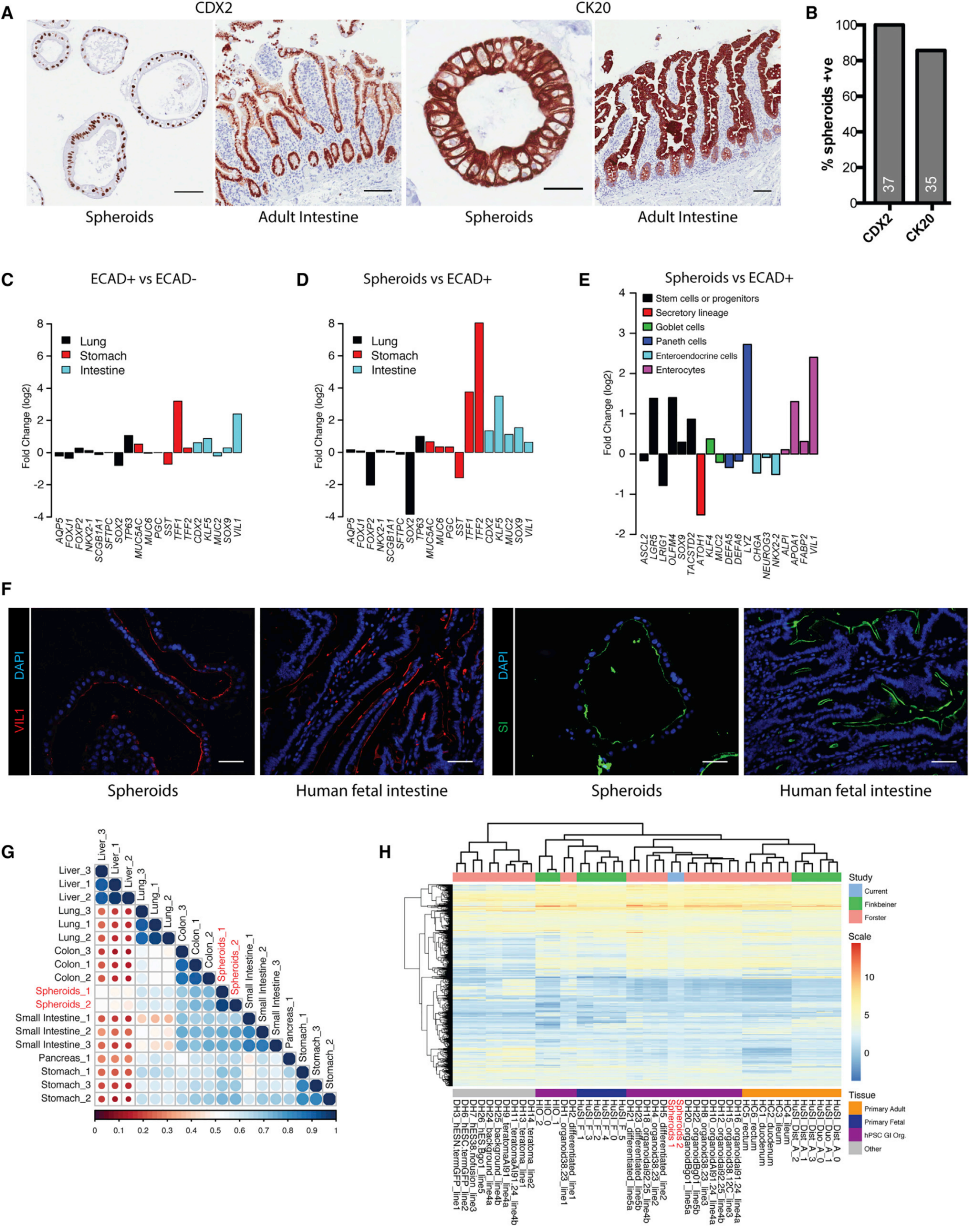
Spheroids Express Markers of Intestinal Epithelial Cell Types and Are Similar in Transcriptome to Primary Human Intestine (A) Representative immunohistochemical staining of spheroids and human adult intestine for CDX2 and CK20. (B) Proportion of spheroids expressing CDX2 and CK20; numbers at the bottom of each bar denote total number of spheres counted. (C) Relative transcript expression levels of markers of lung, stomach, and intestine for ECAD^+^ cells relative to ECAD^−^ cells (n = 3 biological replicates, 1 from H1 hESCs and 2 from H9 hESCs). (D) Relative transcript expression levels of markers of lung, stomach, and intestine for spheroids relative to ECAD^+^ cells. (E) Relative transcript expression levels of markers of intestinal epithelial cell types for spheroids relative to ECAD^+^ cells. Data in (D) and (E) are for spheroids derived from H1 hESCs. (F) Representative immunofluorescence staining of spheroids and human fetal small intestine shows expression of brush border markers VIL1 and SI. (G) Correlation plot contrasting the gene expression profile of H1 hESC-derived spheroids with human adult organs. (H) Gene expression clustergram comparing H1 hESC-derived spheroids with other hPSC-derived intestinal organoids and human fetal and adult intestinal tissues. Scale bars, 100 μm (A) and 50 μm (F). See also Figure S3.

E-cadherin expression is not restricted to a particular cell lineage during development, so we next sought to clarify the identity of the spheroids generated from ECAD^+^ cells. A mixture of cells expressing CDX2 or NKX2.1 were present within the endoderm-enriched 3D tissues used to isolate the ECAD^+^ cells; however, immunohistochemistry revealed that all assayed spheroids expressed the intestinal transcription factor CDX2 (Figures 3A and 3B). No spheroids were observed that expressed NKX2-1 (Figure S3A) or SOX2 (data not shown). Almost all spheroids also robustly expressed cytokeratin 20 (CK20), which is expressed in mature intestinal and gastric epithelium (Moll et al., 1990), as well as SOX9, which is found within the proliferating compartment of the intestine (Figures 3A, 3B, and S3A). These data show that the ECAD^+^ population generated spheroids with features consistent with a gastrointestinal lineage.

Strikingly, this outcome was obtained by culturing cells in growth medium that is not optimized for gastrointestinal organoid growth (Sato et al., 2011). Omitting specific MTEC medium constituents during ECAD^+^ cell co-culture with HLFs was performed to identify key components. Insulin-transferrin-selenium (ITS) and bovine pituitary extract (BPE) have been used as mitogenic supplements in low-serum media and intestinal epithelial cultures (Baten et al., 1992, Jumarie and Malo, 1991, Takenaka et al., 2014), but only ITS removal significantly reduced both spheroid number and size (Figures S3B and S3C). Removal of epidermal growth factor (EGF) and retinoic acid (RA) from MTEC media also had no visible effect (data not shown). Finally, addition of Y-27632 for the duration of spheroid culture, to inhibit the rho-associated protein kinase (ROCK) pathway, which is known to promote survival of single cells and cloning efficiency (Watanabe et al., 2007), almost doubled the spheroid-forming frequency to 1:100 cells in MTEC medium without affecting size (Figures S3B and S3C).

For further characterization, we performed global mRNA expression profiling on the ECAD+ and ECAD^−^ populations isolated from the 3D endoderm-derived tissues at day 15, as well as spheroids isolated at day 20 of culture. We first assessed the similarity of ECAD^+^ cells, ECAD^−^ cells, and spheroids to *in vivo* tissues by contrasting them with custom gene sets comprising EMAPA ontology and single-cell RNA-sequencing data from primary adult intestinal organoids (Grün et al., 2015). Gene set enrichment mapping of our gene expression profiling data onto the custom gene set revealed that a broad range of processes associated with organ development and morphogenesis were enriched in the ECAD^+^ cells over the ECAD^−^ cells (Figure S3F), supporting their organ precursor status. Also, the transcript levels of markers associated with intestine or stomach development were enriched in the ECAD^+^ cells when compared with the ECAD^−^ cells, but lung-related transcripts were not (Figure 3C). Analysis of the spheroids over ECAD^+^ cells demonstrated increased expression of intestine and stomach-associated genes, but not lung (Figure 3D). Gene set enrichment of the spheroids over ECAD^+^ cells highlighted the intestinal identity of the spheroids, displaying enrichment for nodes representative of general intestinal development, as well as specific intestinal epithelial cell types (Figure S3G). Indeed, markers of enterocytes (*VIL1*, *APOA1*, *FABP2*) and intestinal stem cells (*LGR5*, *OLFM4*, *TACSTD2*) were all elevated in the spheroids when contrasted with the ECAD^+^ cells (Figure 3E). However, markers of goblet cells (*MUC2*), enteroendocrine cells (*CHGA*, *NEUROG3*, *NKX2-2*) and general commitment to the secretory lineage (*ATOH1*) were depleted (Figure 3E). Immunofluorescence staining for intestinal brush border markers villin (VIL1) and the digestive enzyme sucrose-isomaltase (SI) demonstrated expression and protein localization consistent with that observed in human fetal small intestine (Figure 3F). A combination of CDX2, VIL1, and SI is restricted to the small intestine (Beaulieu et al., 1990, Gorvel et al., 1991, Moskaluk et al., 2003), providing a regional identity for the spheroids.

Finally, we assayed the similarity of the ECAD cells and spheroids to other tissues. Principal component analysis of the transcript profiles of hESCs, ECAD^−^ and ECAD^+^ cells, and spheroids demonstrated that the ECAD^−^ and ECAD^+^ samples showed large variance with undifferentiated hESCs, but that the ECAD^+^ cells were closest to the spheroids in the second principal component (Figure S3E). Comparison of the spheroid expression profiles with those of endoderm-derived human adult organs (liver, lung, pancreas, stomach, small intestine, and colon) revealed the highest similarity to gastrointestinal tissues (Figure 3G). We obtained expression profiles for hPSC-derived intestinal organoids produced by others (Finkbeiner et al., 2015, Forster et al., 2014, Spence et al., 2011), as well as the control human adult and fetal intestinal tissues included in these studies, and contrasted them with our spheroids. The *in vitro* methodology utilized to generate the hPSC-derived intestinal organoids described in Spence et al. (2011) produces tissues that most closely resemble human fetal intestinal samples (Finkbeiner et al., 2015), but a period of *in vivo* engraftment is sufficient to produce a more mature phenotype (Finkbeiner et al., 2015, Watson et al., 2014). The study by Forster et al. (2014) demonstrated that LGR5^+^ intestinal progenitors could be isolated from teratomas produced by *in vivo* engraftment of LGR5-GFP reporter hPSCs, and that LGR5^+^ cells isolated from these teratomas formed intestinal organoids that displayed more mature properties (Forster et al., 2014). Comparison with these organoid and primary human datasets demonstrated that the ECAD^+^ cell-derived spheroids clustered closely with the more mature Forster organoids (Figure 3H), which together more closely resembled primary human adult intestinal tissues than fetal intestinal tissues.

Together these data support a small intestinal identity for the ECAD^+^ cell-derived spheroids, and show that they retain properties that are similar to hPSC-derived intestinal tissues that have undergone *in vivo* engraftment. The cystic morphology and expression of some intestinal epithelial markers in the spheroids resemble primary enterospheres (Stelzner et al., 2012), so herein they are referred to as hPSC-derived enterospheres (hEnS).

### Growth and Differentiation of hEnS in Different Media Conditions

**Figure 4:**
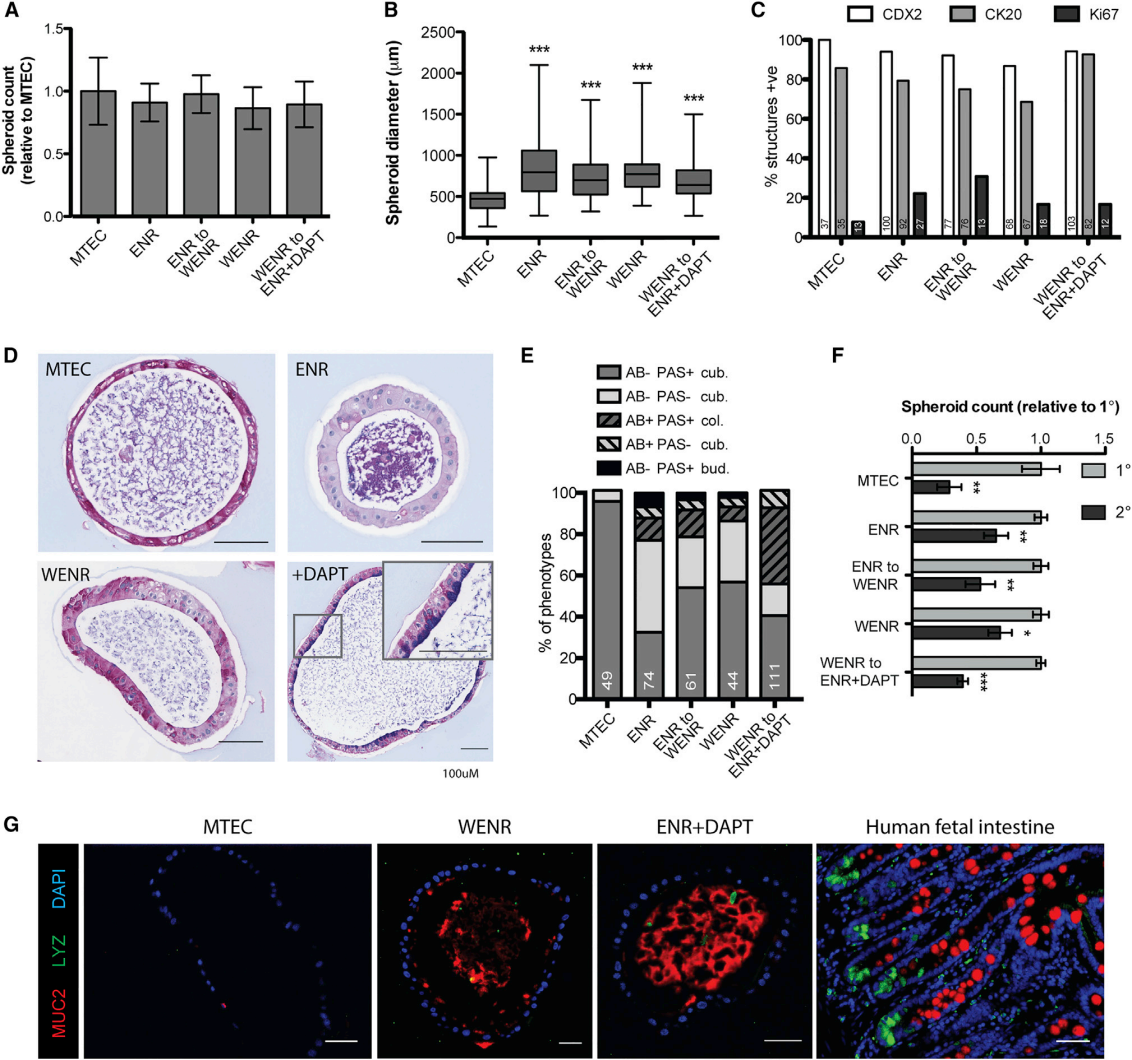
Comparison of hEnS Phenotypes Produced in Different Media Conditions (A) Comparison of number of hEnS produced in different media conditions (mean ± SEM, n ≥ 3 independent experiments). (B) Comparison of diameter of hEnS produced in different media conditions (mean ± SEM, n ≥ 40 spheroids spanning 3 independent experiments; ^∗∗∗^p < 0.0001). (C) Proportion of hEnS expressing CDX2, CK20, and Ki67; numbers at the bottom of each bar denote total number of spheres counted. (D) Representative Alcian blue and periodic acid-Schiff (AB-PAS) staining of hEnS; most prevalent phenotype displayed for each media condition. (E) Proportion of phenotypes identified by AB-PAS staining per tissue section; numbers at the bottom of each bar denote total number of spheres counted in sections. (F) Number of secondary spheroids produced relative to primary for corresponding media condition (mean ± SEM, n = 3 independent experiments; ^∗^p = 0.0142, ^∗∗^p < 0.01, ^∗∗∗^p < 0.0001). (G) Representative immunofluorescence staining for LYZ and MUC2 of hEnS in different media conditions and human fetal small intestine. All data shown are for hEnS derived from H1 hESCs. Scale bars, 100 μm (D) and 50 μm (G). See also Figure S4.

The derivation and culture of primary human GI organoids requires a cocktail of factors such as WNT3a, EGF, Noggin, and R-Spondin (McCracken et al., 2014, Mustata et al., 2013, Sato et al., 2009, Sato et al., 2011), most of which are absent in MTEC medium. Therefore, widely used intestinal-specific media conditions were utilized to determine whether structures that were more representative of mature intestinal epithelium could be generated. ECAD^+^ cells cultured in intestinal medium containing EGF, Noggin, and R-Spondin (ENR) gave rise to a similar number of hEnS (Figure 4A), but those that did form were larger (Figures 4B and S4) and expressed CK20 in similar numbers as MTEC spheroids (Figure 4C). Combined periodic acid-Schiff (PAS) and Alcian blue (AB) staining discriminates glycoproteins and neutral mucins (stained magenta) from acid mucins (blue), with the latter present in functional goblet cells. MTEC medium produced homogeneous hEnS, with >90% composed of PAS^+^ AB^−^ cuboidal epithelium. ENR medium resulted in greater heterogeneity, with ∼40% of spheroids displaying a PAS^−^ AB^−^ phenotype (Figures 4D and 4E), and small numbers of AB^+^ structures indicative of secretory lineages. The addition of WNT3a to ENR is thought to induce intestinal maturation in culture (Fordham et al., 2013). Similar hEnS numbers, sizes, and percentage of structures expressing CDX2 or CK20 seen in ENR were observed when ENR was supplemented with WNT3a halfway through the 20-day culture period (ENR-to-WENR) or when WNT3a was supplemented from the start (WENR) (Figures 4A–4C). Both ENR-to-WENR and WENR conditions produced hEnS similar to ENR alone, but with a reduction in the fraction of PAS^−^ AB^−^ structures (Figures 4D and 4E). The γ-secretase inhibitor DAPT ((S)-tert-butyl 2-((S)-2-(2-(3,5-difluorophenyl)acetamido)propanamido)-2-phenylacetate) inhibits NOTCH signaling, promoting goblet cell differentiation in intestinal tissues (van Es et al., 2005, Forster et al., 2014, Mustata et al., 2013). Culturing ECAD^+^ cells in WENR for 10 days, followed by withdrawal of WNT3a and addition of DAPT (WENR-to-ENR + DAPT) for 10 days, produced approximately the same, but larger, structures than MTEC, but nearly all expressed CDX2 and CK20 (Figures 4A–4C). The WENR-to-ENR + DAPT condition also elicited almost 40% of hEnS to express acid mucins (AB^+^) in a pattern consistent with a goblet cell phenotype (Figures 4D and 4E). Immunofluorescence staining demonstrated that MTEC spheroids did not express MUC2 or the marker lysozyme (LYZ) at the protein level; however, spheroids expressing MUC2 and LYZ could be observed in all of the intestine-specific media conditions (Figure 4G and data not shown).

Secondary spheroid formation and long-term propagation indicate the presence of functional stem cells, so we tested the capacity of the different media to enable passaging of hEnS. MTEC medium formed hEnS in 2° assays, but efficiency was less than half that of 1° MTEC hEnS formation (Figure 4F). Insufficient hEnS formed in 3° assays in MTEC medium for continued passage (data not shown). Significantly lower 2° hEnS formation efficiencies were observed for ENR, WENR, WENR-to-ENR, and WENR-to-ENR + DAPT (Figure 4F), showing that they were not suitable for long-term propagation.

### Long-Term Propagation of hEnS

**Figure 5:**
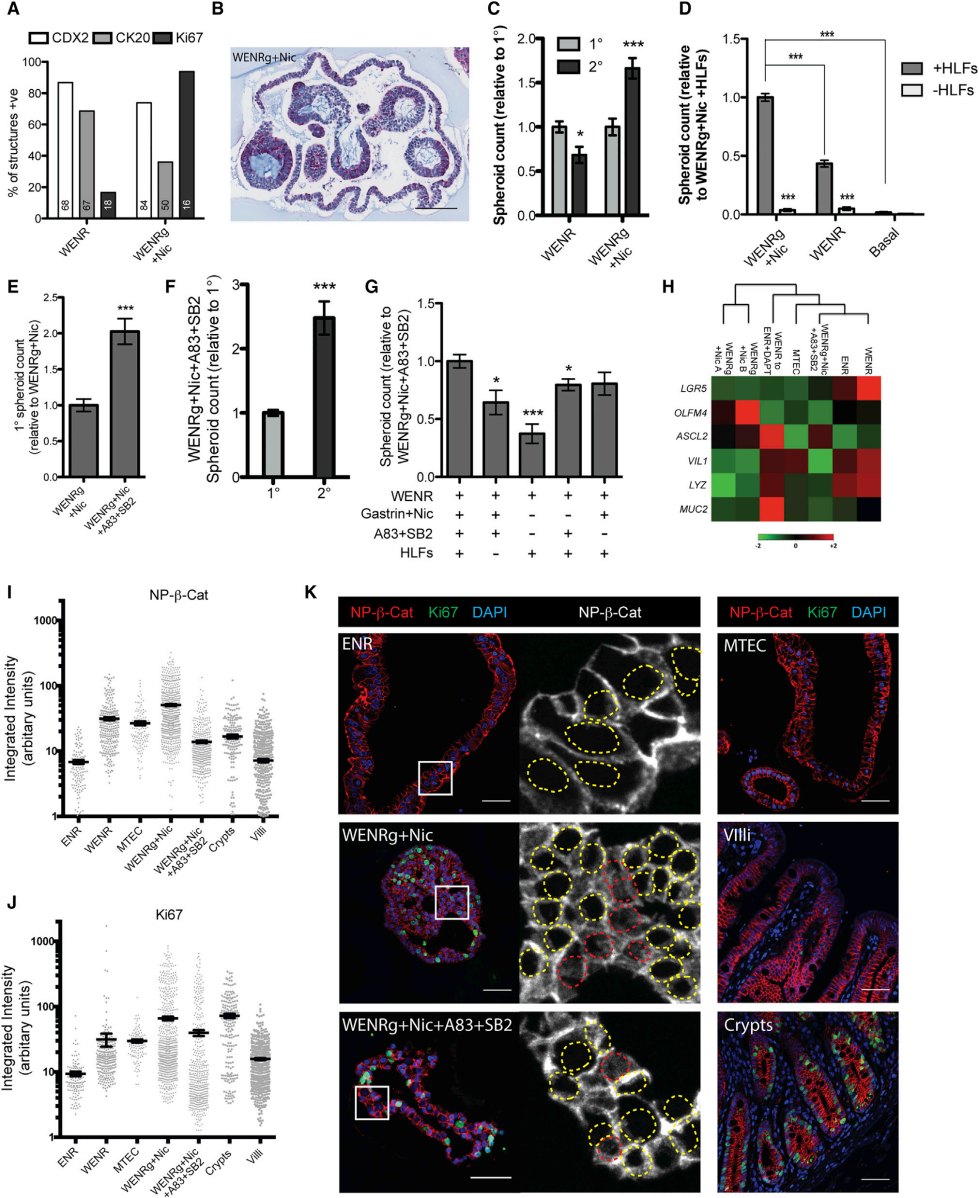
Long-Term Culture Requirements of hEnS in Specific Media Conditions and Their Phenotypic Properties (A) Comparison of the proportion of hEnS expressing CDX2, CK20, and Ki67 in WENR and WENRg + Nic media; numbers at the bottom of each bar denote total number of spheres counted. (B) Representative AB-PAS staining of complex budding structures produced in WENRg + Nic. (C) Number of secondary spheroids produced relative to primary in WENR and WENRg + Nic (mean ± SEM, n = 3 independent experiments; ^∗^p = 0.0142, ^∗∗∗^p = 0.0008). (D) Number of hEnS produced upon passaging in WENRg + Nic, WENR, and basal media with or without support cells (mean ± SEM, n = 3 independent experiments; ^∗∗∗^p < 0.0001). (E) Comparison of the relative number of hEnS produced in WENRg + Nic and WENRg + Nic + A83 + SB2 media (mean ± SEM, n = 3 independent experiments; ^∗∗∗^p = 0.0001). (F) Number of secondary spheroids produced relative to primary in WENRg + Nic + A83 + SB2 (mean ± SEM, n = 3 independent experiments; ^∗∗∗^p < 0.0001). (G) Relative number of hEnS produced upon addition or removal of key growth factors or support cells in a WENR media background (mean ± SEM, n = 3 independent experiments; ^∗^p < 0.05, ^∗∗∗^p < 0.0001). (H) Clustergram of intestinal gene expression grouped by type of media condition used to culture hEnS. Non-supervised hierarchical clustering was used to display common gene expression on a heatmap; normalized to *GAPDH* expression (*Z* scores calculated from 2^−ΔCt^ values; n = 2 biological replicates from independent experiments). (I and J) Nuclear intensity of NP-β-catenin (I) and Ki67 staining (J) in hEnS cells from different media conditions (mean ± SEM, n ≥ 120 cells analyzed per condition). (K) Representative immunofluorescence staining for NP-β-catenin and Ki67 in hEnS in different media conditions as well as human fetal small intestine. White boxes indicate zoomed-in regions. Dashed yellow and red borders indicate absence and presence, respectively, of nuclear NP-β-catenin staining. All data shown are for hEnS derived from H1 hESCs. Scale bars, 100 μm (B) and 50 μm (K). See also Figure S5.

Gastrin and nicotinamide (WENRg + Nic) have been shown to improve plating efficiency and long-term maintenance of primary human intestinal organoids (Sato et al., 2011), so we tested whether this medium could support hEnS derivation and maintenance. Primary cultures of hEnS grown from H1 hESC-derived ECAD^+^ cells in WENRg + Nic were observed at a higher frequency (1:100 cells) and smaller size than WENR (Figures S5A and S5B). The WENRg + Nic hEnS expressed SOX9 (Figure S5C), but only about half as many structures displayed CK20 reactivity as observed in WENR (Figure 5A). Only a few spheroids with AB^+^ cells were present in WENRg + Nic, but about 50% of hEnS were of a more complex “budding” structure that were AB^−^, PAS^+^, and displayed low to no expression of CK20 and CDX2 (Figures 5B, S5D, and S5E). The lack of AB and CK20, as well as low/no CDX2 suggested that they were composed of cells that were less differentiated compared with those produced in other media conditions (Silberg et al., 2000). In support of this, and in contrast to other media conditions tested, WENRg + Nic yielded significantly more 2° hEnS than were produced in 1° derivation assays from ECAD^+^ cells (Figure 5C), and supported their continued passage (more than 8 months; >13 passages) and cryopreservation, avoiding the need to derive *de novo* cultures from hESCs. Primary hEnS from H9 hESCs were produced in WENRg + Nic media at a lower frequency (∼1:2,000 cells) than from H1 hESCs, but could be subsequently expanded upon passaging (data not shown). WENRg + Nic elicited robust expression of the cell proliferation marker Ki67 (Basak et al., 2014), with 90% of WENRg + Nic hEnS containing Ki67^+^ cells, compared with 10%–30% Ki67^+^ for other media (Figures 5A and S5F). Since WENRg + Nic enabled robust hEnS expansion, we evaluated the necessity of the HLF support cells in this condition. Substituting the HLFs with human dermal fibroblasts (HDFs) led to a large decline in hEnS formation efficiency, although, unlike HUVECs (Figure 2D), those hEnS that did form were larger than those found in HLF conditions (Figures S5G–S5I). HLFs removal led to an almost total loss of hEnS formation in WENRg + Nic, WENR, and basal medium, underscoring the necessity of these cells in supporting the propagation of the hEnS (Figure 5D). Inhibition of the transforming growth factor β (TGF-β) and p38 signaling (usually via addition of the small-molecule inhibitors of Alk4/5/7 [A83-01] and p38 [SB202190]) has been demonstrated to overcome the window of growth arrest/crisis that occurs after ∼3 months when adult primary intestinal organoids are grown in WENRg + Nic (Sato et al., 2011). Derivation of 1° hEnS from ECAD^+^ cells in the presence of HLFs was twice as efficient in WENRg + Nic supplemented with A83-01 and SB202190 (WENRg + Nic + A83 + SB2) than for WENRg + Nic only (Figure 5E). Spheroid size was smaller in the presence of the inhibitors, but complex budding structures were present at a frequency similar to that observed in WENRg + Nic (Figures S5B, S5D, and S5I). The frequency of Ki67^+^ hEnS in the presence of the inhibitors was similar to that of WENRg + Nic without inhibitors, but the fraction of CDX2+ hEnS was substantially reduced (Figures S5J and S5L). Transfer of hEnS established and propagated in WENRg + Nic with HLFs for more than 10 passages to WENRg + Nic + A83 + SB2 with HLFs yielded a 2-fold increase in hEnS-forming efficiency (Figure S5K). hEnS derived in WENRg + Nic + A83 + SB2 with HLFs generated 2-fold more hEnS in 2° assays, elevating the hEnS-forming efficiency to ∼1:30 cells (Figure 5F). Growth of hEnS in WENRg + Nic was reliant upon the presence of HLFs (Figure 5D); however, hEnS previously cultured in WENRg + Nic + A83 + SB2 formed 2° hEnS in the absence of feeders, albeit with a moderate reduction in efficiency (Figure 5G). Previous culture with the inhibitors inured the 2° hEnS formation efficiency to removal of gastrin + nicotinamide or A83-01 + SB202190, but not both, showing that sustained increases in efficiency were not mediated solely by the presence of the inhibitors.

Next, we assayed how the different media conditions influenced the expression of key intestinal stem cell and differentiation markers. hEnS generated in ENR, WENR, and WENR-to-ENR + DAPT expressed higher levels of *VIL1*, *LYZ*, and *MUC2* (Figure 5H), implying a more differentiated phenotype. hEnS grown in WENRg + Nic clustered separately and expressed higher levels of the stem cell markers *OLFM4* and *ASCL2*, whereas WENRg + Nic + A83 + SB2 had relatively lower OLMF4 and higher LGR5. hEnS grown in WENR-to-ENR + DAPT showed the highest expression of *MUC2*.

Finally, we tested how media conditions might be eliciting altered hEnS behavior. Since Wnt signaling is recognized to be the dominant mechanism for driving the proliferation of intestinal stem cells (Fevr et al., 2007, Krausova and Korinek, 2014), we assayed levels of the active form of the Wnt signal mediator β-catenin. Phosphorylation at Ser33/37/Thr41 by GSK-3 inactivates β-catenin (Yost et al., 1996), so we looked at active, nuclear β-catenin using an antibody that recognizes only the non-phosphorylated form (NP-β-catenin). Membrane-bound NP-β-catenin was observable in all conditions tested. Nuclear NP-β-catenin was most frequent in the WENRg + Nic, WENR, and MTEC conditions (Figures 5I–5K), but levels for ENR were much lower, and similar to those observed in the human intestinal villi. WENRg + Nic + A83 + SB2 displayed nuclear NP-β-catenin levels that were lower than for WENRg + Nic alone, but were more equivalent to those seen in the human crypts, despite all three samples displaying higher Ki67 expression levels than other conditions (Figures 5I and 5J).

Collectively, these observations demonstrate that specific culture conditions allow for the long-term culture of hEnS, and that the most efficient culture conditions elicit Wnt signaling levels that match those observed in the intestinal crypts.

### Cell-Cycle Analysis of hEnS Equipped with a Fluorescence Cell-Cycle Reporter

**Figure 6:**
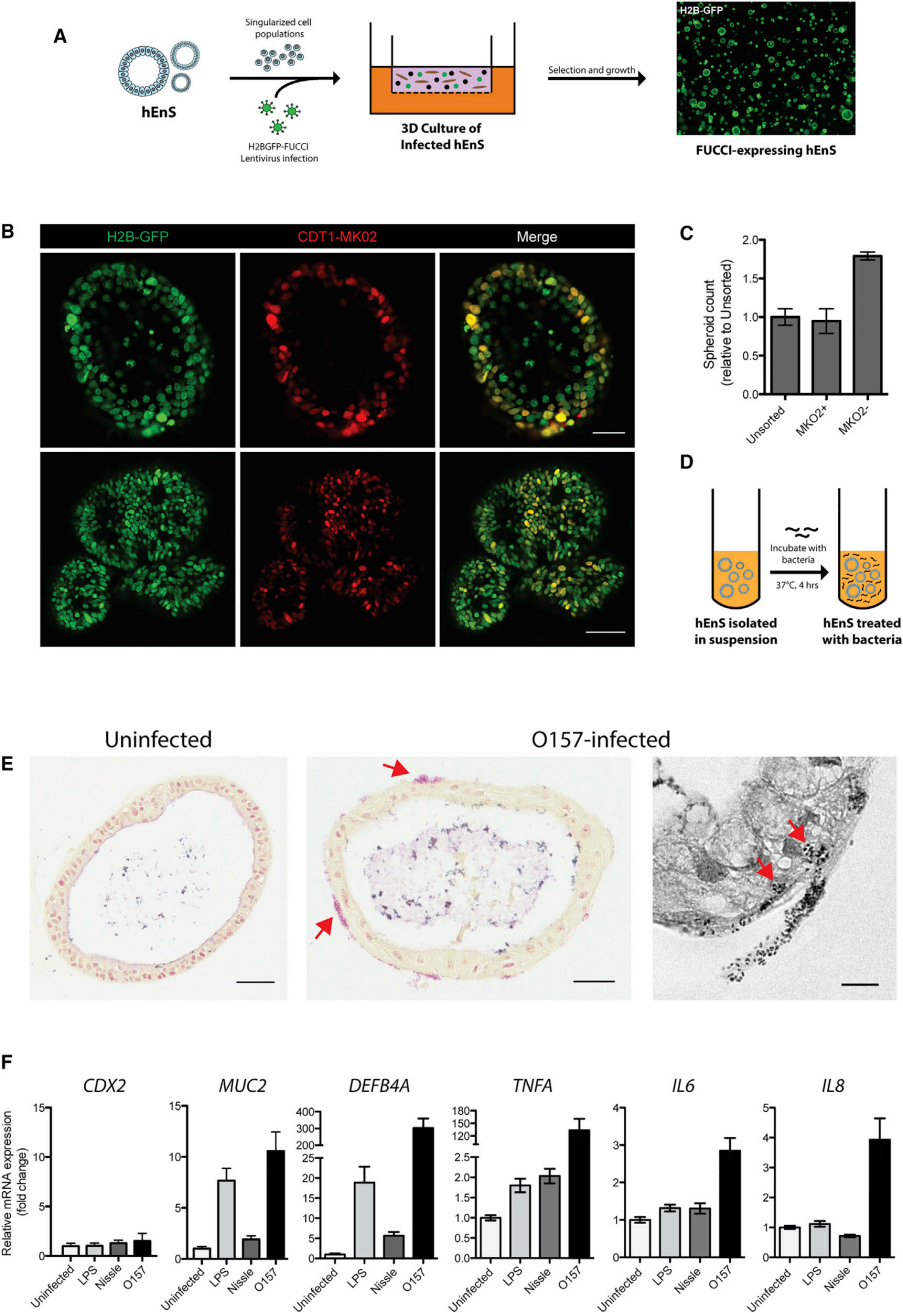
Genetic Modification and Functional Interrogation of hEnS (A) Schematic of infection of hEnS precursor cells with FUCCI lentivirus, and H2B-GFP photograph showing pure population of FUCCI-expressing hEnS achieved after antibiotic selection and propagation. (B) Live fluorescence imaging shows that FUCCI-infected hEnS ubiquitously express H2B-GFP, and cells in G_1_ phase express CTD1-MK02. Scale bars, 50 μm (top) and 100 μm (bottom). (C) Relative number of hEnS propagated from sorted MKO2^+^, MKO2^−^, and unsorted hEnS cells at the same seeding density (mean ± SEM, n = 3 independent wells of an experiment). (D) Schematic of bacterial infection of hEnS. (E) Representative Gram staining of uninfected and *E. coli* O157-infected hEnS; red arrows point to areas of bacterial infiltration. Scale bars, 50 μm (left and center) and 10 μm (right). (F) Relative transcript expression levels of *CDX2*, *MUC2*, *DEFB4A*, *TNF*, *IL6*, and *IL8* in hEnS treated with LPS or bacteria; normalized to *GAPDH* expression (values represent linear fold change; n = 2 independent experiments). All data shown are for hEnS derived from H1 hESCs.

Genetic modification experiments can provide fundamental insights into tissue function. We reasoned that the population of singularized precursor cells used to form hEnS would be amenable to transgenic alteration, avoiding the laborious process of creating stable genetically modified hPSC lines. We took advantage of this utility by infecting dissociated hEnS cells with a lentivirus carrying the H2BGFP-FUCCI reporter (Calder et al., 2013) (Figure 6A). This reporter consists of H2B-GFP, which decorates chromatin, linked via a 2A sequence to mKO2-Cdt1, the expression of which is restricted to cells in G_1_/G_0_ (Sakaue-Sawano et al., 2008). Following puromycin selection, we derived hEnS that ubiquitously expressed H2B-GFP, with cells in G_1_/G_0_ transiently expressing mKO2-Cdt1 (Figure 6B). Flow quantification indicated that ∼92% of cells were mKO2^+^ (data not shown).

We utilized the H2BGFP-FUCCI reporter for cell-cycle analysis of hEnS cells. As described earlier, hEnS produced in WENRg + Nic consist of Ki67-expressing cells, which may be capable of spheroid propagation. Since cells that are actively cycling are more likely to represent proliferative or stem cells (Calder et al., 2013), we hypothesized that the mKO2^−^ cell fraction would be enriched for hEnS-forming stem cells, while the mKO2^+^ fraction, or cells in G_1_/G_0_, would contain a greater proportion of non-proliferative or differentiated cells.

To show proof of principle, we tested this hypothesis by FACS-separating FUCCI-equipped hEnS cells by mKO2-Cdt1 expression, and comparing the frequency at which each fraction generates new hEnS in culture. We found that while mKO2^+^ and unsorted cells produced hEnS in similar numbers, mKO2^−^ cells produced hEnS with almost double the frequency (Figure 6C). This indicates that the occurrence of hEnS-propagating cells was higher in the actively cycling population than in cells in G_1_/G_0_. Therefore, transgenic modification of hEnS with the H2BGFP-FUCCI reporter permitted cell-cycle analysis and enabled further enrichment for hEnS-forming stem cells.

### hEnS Elicit a Functional Innate Immune Response to Bacterial Infection

Establishing robust assays that test the functionality of *in vitro*-derived intestinal tissues is necessary if they are to be used in downstream biomedical applications. We sought to test whether the hEnS provided a model for studying functional gastrointestinal responses. To functionally interrogate the hEnS, we tested their ability to demonstrate an innate immune response to bacterial infection. Mucin glycoproteins such as MUC2, whose expression was detected in hEnS, are the main component of the first barrier encountered by bacteria in the intestine (Lindén et al., 2008), and increased expression of MUC2 is a well-documented innate response by intestinal cell types confronted with pathogenic bacteria (Forbester et al., 2015, Lindén et al., 2008, Möndel et al., 2009, Vora et al., 2004, Xue et al., 2014). We tested the response of hEnS to bacterial interaction by incubating hEnS with either 100 ng/mL bacterial lipopolysaccharide (LPS), the pathogenic *Escherichia coli* strain O157:H7, or the non-pathogenic probiotic strain Nissle 1917 (both bacteria strains at an MOI of ∼1:50) (Figure 6D). The Nissle 1917 strain serves as a negative control, as it contains a defect in LPS biosynthesis that leads to the production of truncated O-antigen polysaccharide chains (Güttsches et al., 2012). After infection, O157:H7 bacteria could be observed in close association with the hEnS (Figure 6E). Relative to uninfected hEnS, transcript levels for genes associated with an innate immune response, including *MUC2*, *DEFB4A*, *TNF*, *IL6*, and *IL8*, were significantly higher in hEnS treated with pathogenic strain O157:H7, but were not significantly changed for non-pathogenic Nissle 1917 (Figure 6F). LPS treatment induced significant changes only in *MUC2*, *DEFB4A*, and *TNF.* No significant changes in *CDX2* levels were detected in any of the treatments, indicating that the transcript changes were not global events. No changes in *MUC2* or *CDX2* transcripts were observed in undifferentiated H1 hESCs for any of the treatments (data not shown), supporting the specificity of this response to bona fide intestinal cell types. Therefore, hEnS elicited a functional innate immune response to treatment with LPS and enteric pathogens.

## Discussion

We have established an *in vitro* method for generating enterospheres, or hEnS, from hPSCs. Intestinal organoids have been generated *in vitro* from hPSCs by endoderm differentiation protocols that produce raised aggregates of mid/hindgut cells on the culture surface at around day 7 (Fordham et al., 2013, Spence et al., 2011, Watson et al., 2014). Subsequent 3D culture of these progenitor units produces complex organoids that contain multiple intestinal cell types organized in a manner similar to that of native intestine. Our approach uses ECAD expression to isolate singularized epithelial progenitors from hPSC-derived 3D tissues comprising a mixture of endoderm lineages generated via a 4-stage, 26-day differentiation process. Subsequent hEnS formation in a 3D growth environment demonstrates the presence of intestinal stem cells within the ECAD^+^ population that display resilient growth in a variety of media conditions.

In our multistage differentiation strategy, we routinely derived monolayer cultures containing CDX2^+^ cells. CDX2 expression was absent at stages 1 and 2, but emerged by the end of stage 3, following treatment consisting of WNT, fibroblast growth factor (FGF), bone morphogenetic protein, and RA signaling. The emergence of CDX2 at stage 3 may be due to exposure to WNT and FGF signaling, which have been utilized in previous work to specify the mid-/hindgut lineage from endoderm (Spence et al., 2011). Despite the extensive presence of CDX2^+^ cells at the end of stage 3 (day 11), we were unable to isolate spheroids by seeding singularized cells into 3D culture conditions, with or without support cells. However, subsequent culture of cell clumps from stage 3 in a 3D growth environment produced complex structures, composed of mesenchymal cells that we showed supported the integrity and growth of epithelial tubules. Isolation of ECAD^+^ epithelial cells from the 3D tissues enabled the formation of hEnS, showing that functional hEnS-forming stem cells were specified within the 3D structures produced in stage 4.

The differentiation of hPSCs often yields tissues that have an immature fetal phenotype, although several studies have shown correlations between extending the duration hPSC-derived cell types are in culture with the extent of maturation achieved (Lundy et al., 2013, Nicholas et al., 2013, Yang et al., 2014, Zhang et al., 2009). *In vivo* engraftment enables hPSC-derived intestinal organoids to manifest features associated with maturation (Finkbeiner et al., 2015, Forster et al., 2014). Although the *in vitro* generation of fetal enterosphere-like structures was previously reported (Fordham et al., 2013), they failed to display robust expression of intestinal epithelial markers. We have shown that our methodology produces hEnS that are more similar in gene expression profile to hPSC-derived intestinal organoids derived via *in vivo* engraftment than to those made by previously described *in vitro* differentiation protocols, and that our hEnS show greater transcriptional similarity to primary adult intestine than to fetal intestine. The hEnS express markers of enterocytes (VIL1), goblet cells (MUC2), mature brush border proteins (CK20 and SI), and the antimicrobial enzyme LYZ. Of note, the detection of SI expression has only been described in hPSC-derived intestinal organoids after a period of maturation by *in vivo* engraftment (Finkbeiner et al., 2015). We were unable to detect other maturation indicators in the hEnS, such as enteroendocrine marker CHGA and ubiquitous OLMF4 expression (data not shown), indicating that the hEnS achieved an intermediate stage of maturation that surpasses previous *in vitro* methods for generating intestinal tissues, but does not fully attain the maturation level observed following *in vivo* engraftment. Cystic spheroids, as opposed to crypt-villus structures or enteroids, represented the most prevalent phenotype. Taken together, the hEnS display hallmarks of proximal intestinal tissue at around embryonic days 16 to 18 in the mouse or 10–12 gestational weeks in the human (Fordham et al., 2013, Mustata et al., 2013).

Strikingly, hEnS can initially form from ECAD^+^ cells in the absence of growth factors thought to be essential for the formation of intestinal or gastric organoids (McCracken et al., 2014, Sato et al., 2009, Sato et al., 2011, Spence et al., 2011), but do require specific media conditions for long-term propagation and differentiation induction. hEnS grown in MTEC display relatively low expression of both intestinal stem and differentiation-related genes, whereas hEnS grown in ENR, WENR, or ENR + DAPT express higher levels. MTEC produces CK20-expressing structures that are devoid of MUC2, suggesting that they may be mainly composed of enterocytes. In the native intestine, Notch inhibition promotes goblet cell differentiation (van Es et al., 2005, Forster et al., 2014, Mustata et al., 2013), and high expression of *MUC2*, along with CK20 and AB positivity in ENR + DAPT, recapitulates this phenomenon. Since expansion of intestinal organoids is a qualitative measure for the presence of intestinal stem cells, we profiled conditions that might enable long-term propagation of the hEnS. We found that WENRg + Nic facilitated long-term passaging, but unlike the primary adult intestinal organoids reported by Sato et al. (2011), we did not see a window of arrest/crisis for the hEnS when cultured in this medium. We showed that this difference was mediated entirely by the presence of the HLFs in our culture system, which outperformed other support cells in maintaining hEnS. Addition of the Alk4/5/7 (A83-01) and p38 (SB202190) inhibitors to WENRg + Nic not only improved hEnS-forming efficacy but also enabled feeder-free growth. The observation that WENRg + Nic + A83 + SB2 allows robust, feeder-free growth of the hEnS, and that it produces nuclear β-catenin levels similar to those of endogenous crypts is intriguing. WENRg + Nic supplemented with feeders generated the highest levels of nuclear β-catenin, but cultures expanded at half the rate and quickly crashed upon feeder removal. These observations may be related to the “just-right” model of Wnt signaling (Albuquerque et al., 2002, Cadigan and Peifer, 2009), which proposes that optimal Wnt signaling levels, but not levels too high or too low, enable Wnt-driven expansion. Our findings suggest that correct Wnt signaling levels may also be required for the robust *in vitro* expansion of intestinal organoids. Finally, expression of the adult stem cell marker *LGR5* was highest in WENR, suggesting that hEnS grown in this condition may have the most mature phenotype (Fordham et al., 2013, Forster et al., 2014). Although *LGR5* is a Wnt target gene, LGR5 expression was depleted in hEnS cultured in WENRg + Nic relative to WENR or WENRg + Nic + A83 + SB2, implying that the modulation of LGR5 expression is influenced by other interactions.

Generating populations of organoids with homogeneous structure and cell-type composition simplifies the interpretation of cell-specific responses and enables consistency in applications such as screening, where similarity in material across replicate wells is desirable. The more variable and complex intestinal organoids reported by others contain multiple cell types, including mesenchymal components, and are less amenable to high-throughput assays. The method we used produces functional intestinal organoid units that can be utilized in spheroid-forming assays and can be enumerated by automated imaging of calcein green (data not shown). In combination with the long-term propagation potential of hEnS, these spheroids represent attractive screening tools for exploring human-specific gastrointestinal biology. Indeed, although the activation of an innate immune response by the hEnS after treatment with LPS and enteric pathogens does not describe the absolute maturation status of the hEnS, it does demonstrate important aspects concerning their utility: first, they contain functional intestinal cell types that respond to external stimuli in a manner characteristic of native intestinal tissues; and second, they recapitulate aspects of gastrointestinal biology.

Our work establishes hEnS as an *in vitro* model system for studying human intestinal biology, development, and disease.

## Experimental Procedures

### Maintenance and Differentiation of hESCs

H1 and H9 wild-type hESCs (Wicell Research Institute) were cultured on Matrigel (Corning; #354234) in mouse embryonic fibroblast-conditioned medium (MEF-CM) as previously described (Tomishima, 2008). For details of this and all other procedures, see Supplemental Experimental Procedures.

## Author Contributions

R.R.N. and J.S.D. conceived and designed the study, prepared the figures, and wrote the manuscript. R.R.N., S.A., S.B., and J.T.L. performed experimental work. R.R.N., S.A., B.J.C., and J.S.D. analyzed the data. B.J.C. and M.G.S. gave conceptual advice. J.S.D. supervised the project.

## Accession Numbers

The NCBI GEO accession number for the microarray data reported in this paper is GEO: GSE89254.

## Supplemental Information

Document S1. Supplemental Experimental Procedures and Figures S1–S5

Movie S1. Self-Organization of Stage 3 Cells into Epithelial Buds in Stage 4 3D Matrigel-Based Culture Conditions, Related to Figure 1 Images were taken every 6 hr for a total of 8 days. Acquired using a Nikon BioStation CT. Scale bar, 100 μm.

Document S2. Article plus Supplemental Information
