# Supplementary material for: Functional Enterospheres Derived In Vitro from Human Pluripotent Stem Cells
Source: Stem Cell Reports. 2017 Aug 31;9(3):897–912. doi: 10.1016/j.stemcr.2017.07.024 (PMC5599260; doi:10.1016/j.stemcr.2017.07.024)
Supplement: Document S1. Supplemental Experimental Procedures and Figures S1–S5 [file mmc1.pdf]

Stem Cell Reports, Volume *9*

Supplemental Information

Functional Enterospheres Derived *In Vitro* from Human Pluripotent Stem Cells

Rohan R. Nadkarni, Soumeya Abed, Brian J. Cox, Sonam Bhatia, Jennifer T. Lau, Michael G. Surette, and Jonathan S. Draper

Figure S1

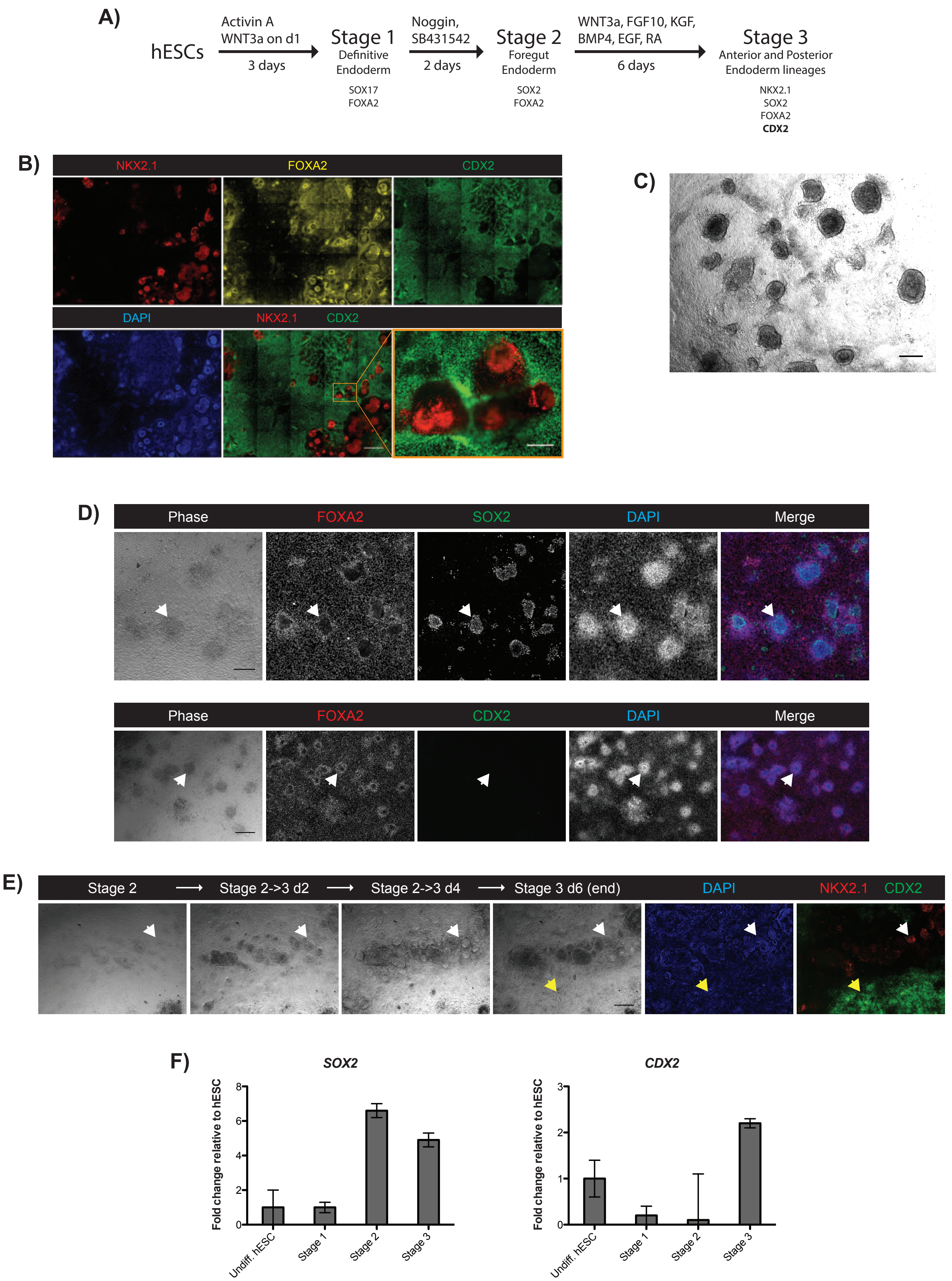

A)
hESCs
Activin A
WNT3a on d1
3 days
Stage 1
Definitive Endoderm
SOX17
FOXA2
Noggin,
SB431542
2 days
Stage 2
Foregut Endoderm
SOX2
FOXA2
WNT3a, FGF10, KGF,
BMP4, EGF, RA
6 days
Stage 3
Anterior and Posterior Endoderm lineages
NKX2.1
SOX2
FOXA2
CDX2
B)
NKX2.1
FOXA2
CDX2
DAPI
NKX2.1 CDX2
C)
D)
Phase
FOXA2
SOX2
DAPI
Merge
Phase
FOXA2
CDX2
DAPI
Merge
E)
Stage 2
Stage 2->3 d2
Stage 2->3 d4
Stage 3 d6 (end)
DAPI
NKX2.1 CDX2
F)
SOX2
Fold change relative to hESC
8
6
4
2
0
Undiff. hESC
Stage 1
Stage 2
Stage 3
CDX2
Fold change relative to hESC
3
2
1
0
Undiff. hESC
Stage 1
Stage 2
Stage 3

Figure S2

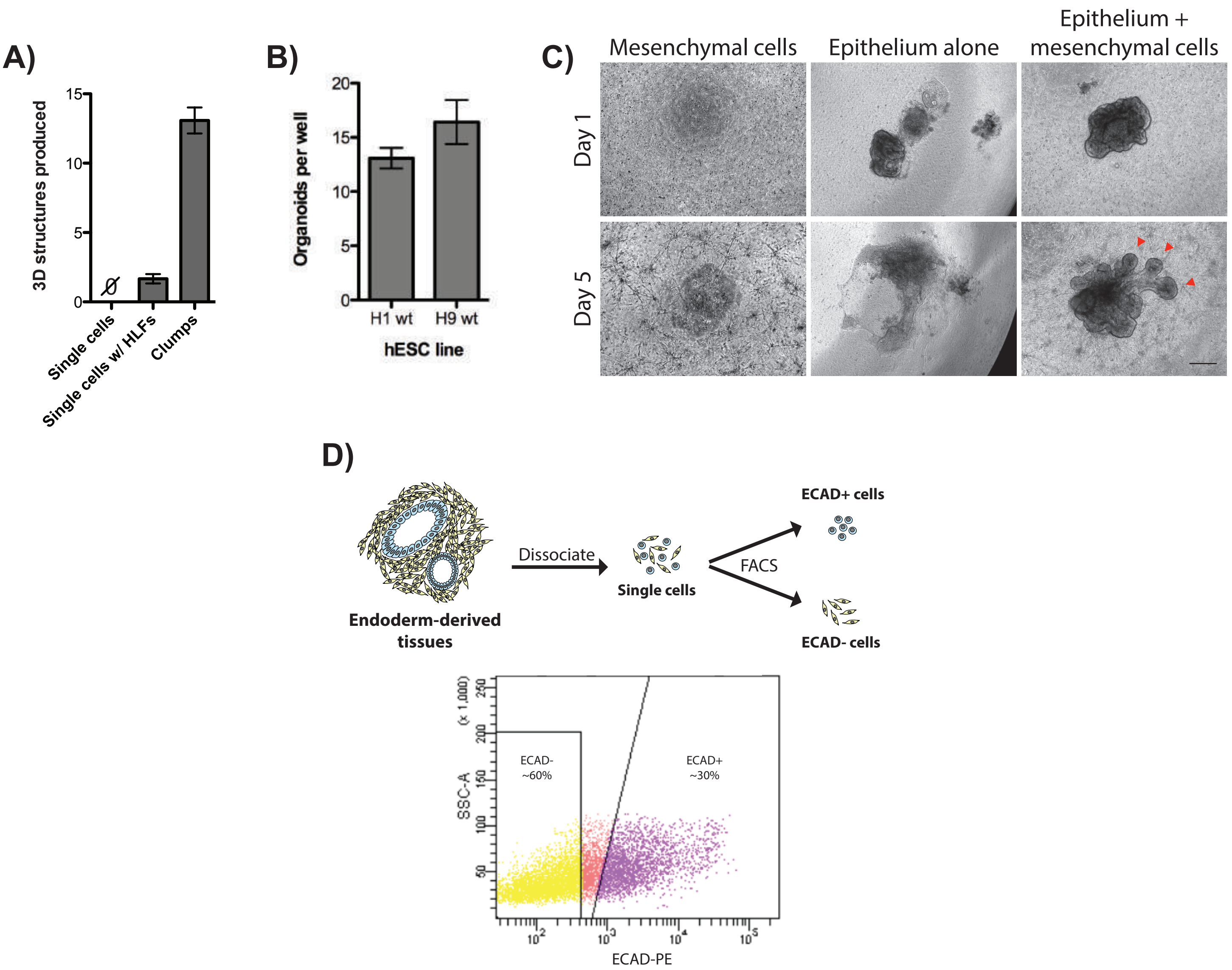
A)
3D structures produced
15
10
5
0
Single cells
Single cells w/ HLFs
Clumps
B)
Organoids per well
20
15
10
5
0
H1 wt
H9 wt
hESC line
C)
Mesenchymal cells
Epithelium alone
Epithelium + mesenchymal cells
Day 1
Day 5
D)
Endoderm-derived tissues
Dissociate
Single cells
FACS
ECAD+ cells
ECAD- cells
SSC-A
(x 1,000)
250
200
150
100
50
ECAD-
~60%
ECAD+
~30%
ECAD-PE

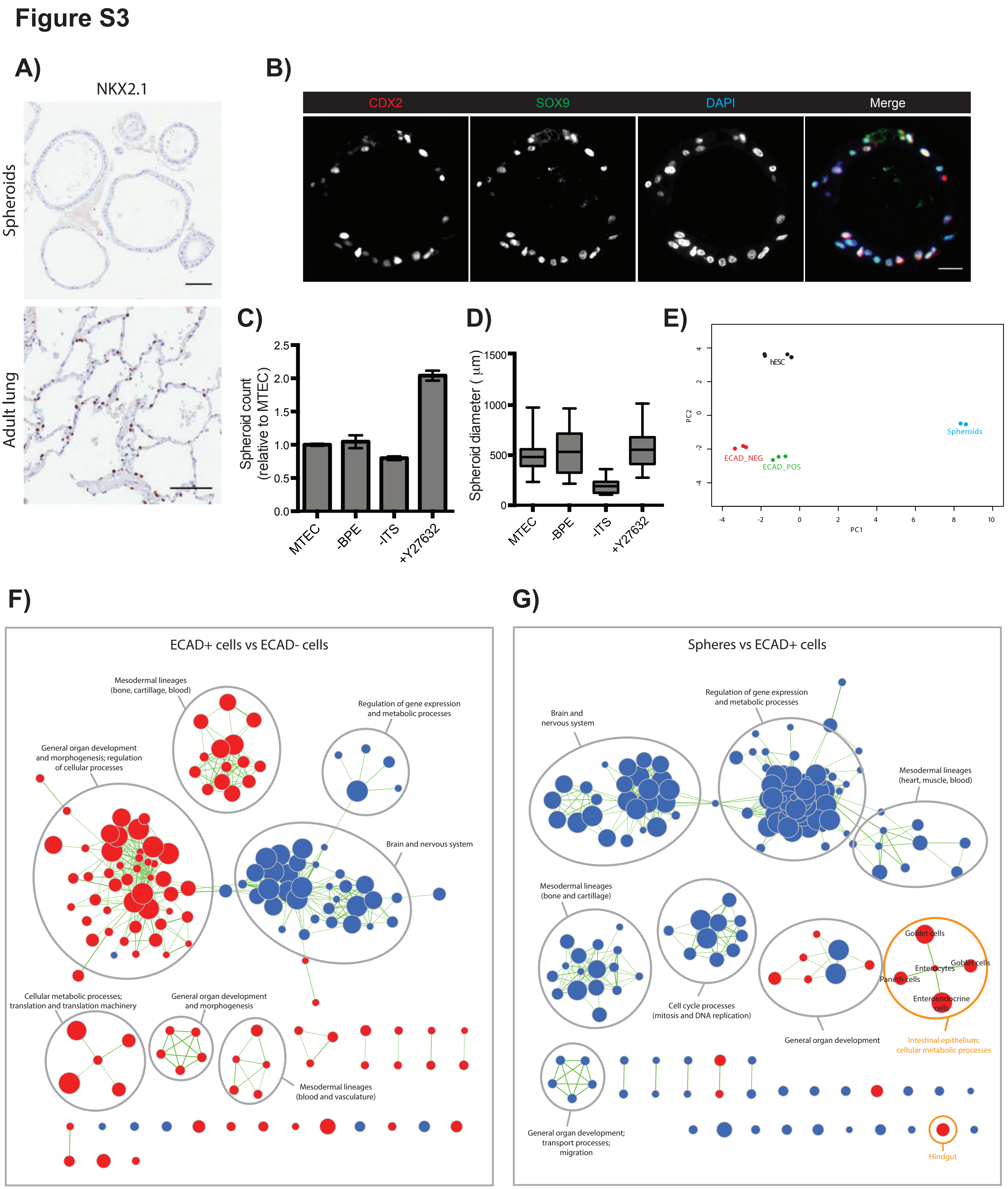

Figure S3
A)
NKX2.1
Spheroids
Adult lung
B)
CDX2
SOX9
DAPI
Merge
C)
Spheroid count
(relative to MTEC)
2.5
2.0
1.5
1.0
0.5
0.0
MTEC
-BPE
-ITS
+Y27632
D)
Spheroid diameter (µm)
1500
1000
500
0
MTEC
-BPE
-ITS
+Y27632
E)
hESC
Spheroids
ECAD_NEG
ECAD_POS
PC2
PC1
F)
ECAD+ cells vs ECAD- cells
Mesodermal lineages
(bone, cartillage, blood)
Regulation of gene expression
and metabolic processes
General organ development
and morphogenesis; regulation
of cellular processes
Brain and nervous system
Cellular metabolic processes;
translation and translation machinery
General organ development
and morphogenesis
Mesodermal lineages
(blood and vasculature)
G)
Spheres vs ECAD+ cells
Regulation of gene expression
and metabolic processes
Brain and
nervous system
Mesodermal lineages
(heart, muscle, blood)
Mesodermal lineages
(bone and cartillage)
Cell cycle processes
(mitosis and DNA replication)
General organ development
Goblet cells
Goblet cells
Enterocytes
Paneth cells
Enteroendocrine
cells
Intestinal epithelium;
cellular metabolic processes
General organ development;
transport processes;
migration
Hindgut

Figure S4

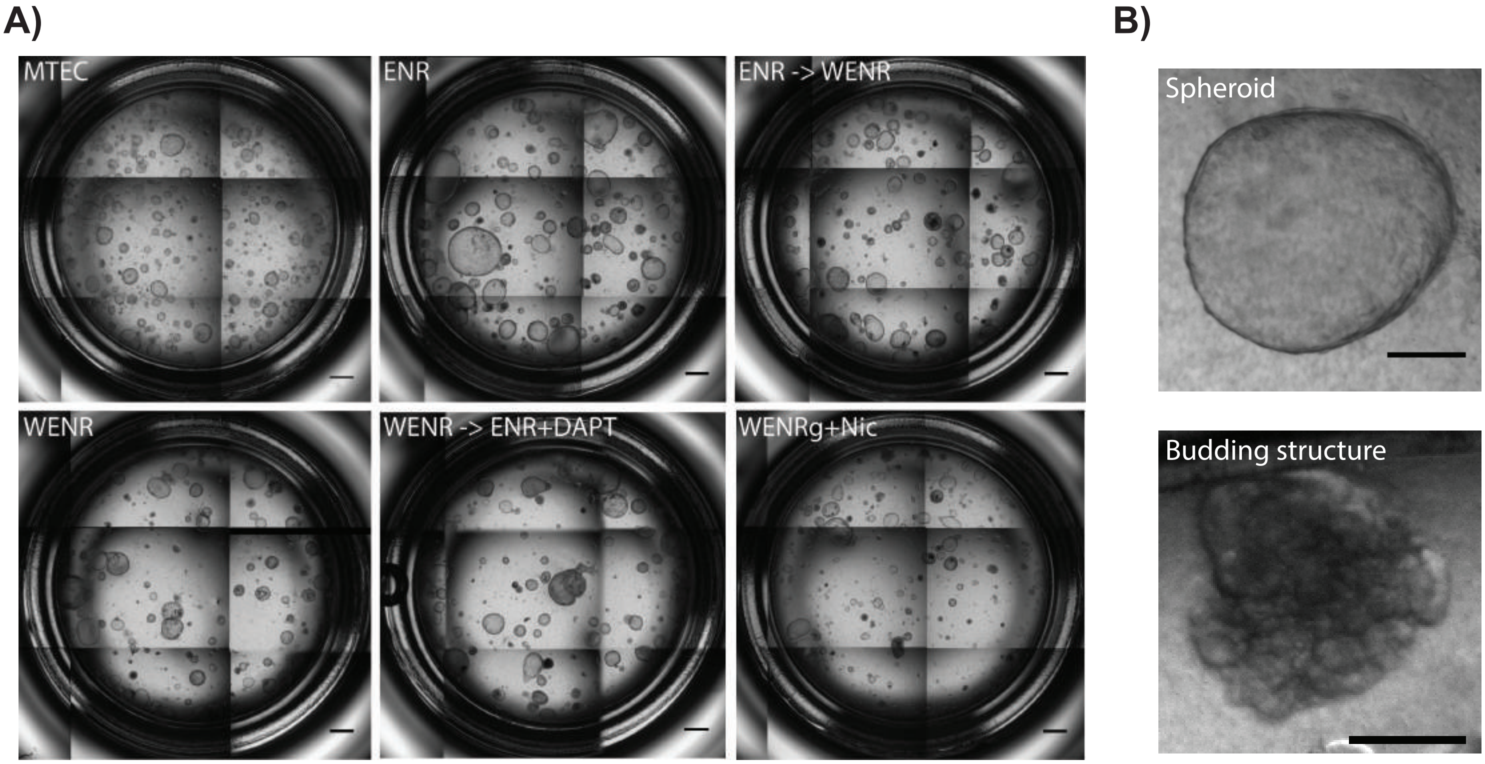
A)
MTEC
ENR
ENR -> WENR
WENR
WENR -> ENR+DAPT
WENRg+Nic
B)
Spheroid
Budding structure

Figure S5

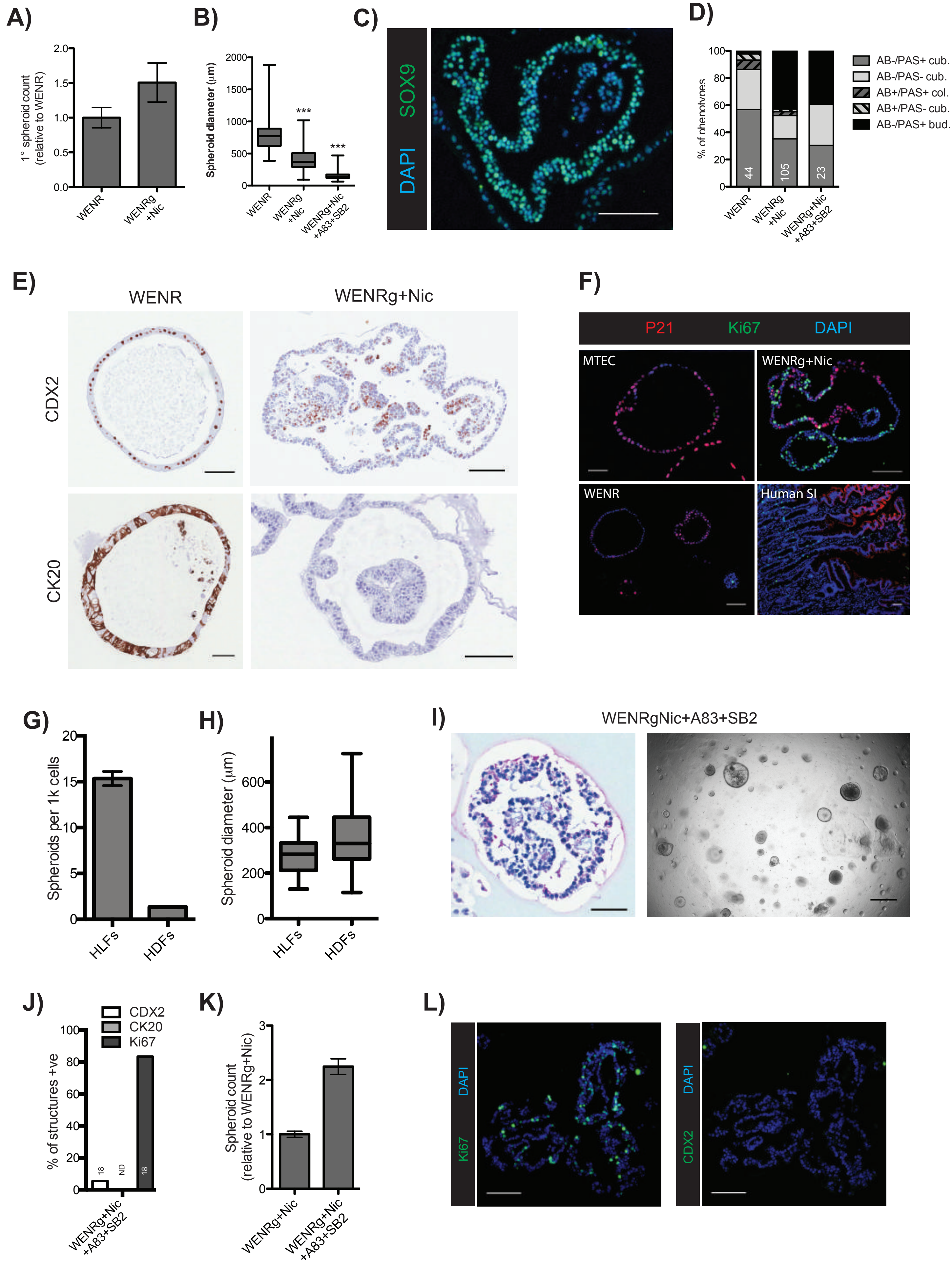

A)
1° spheroid count (relative to WENR)
WENR
WENRg +Nic
B)
Spheroid diameter (µm)
***
WENR
WENRg +Nic
WENRg+Nic +A83+SB2
C)
DAPI
SOX9
D)
% of phenotypes
44
105
23
AB-/PAS+ cub.
AB-/PAS- cub.
AB+/PAS+ col.
AB+/PAS- cub.
AB-/PAS+ bud.
E)
WENR
WENRg+Nic
CDX2
CK20
F)
P21
Ki67
DAPI
MTEC
WENRg+Nic
WENR
Human SI
G)
Spheroids per 1k cells
HLFs
HDFs
H)
Spheroid diameter (µm)
HLFs
HDFs
I)
WENRgNic+A83+SB2
J)
CDX2
CK20
Ki67
% of structures +ve
18
ND
18
WENRg+Nic +A83+SB2
K)
Spheroid count (relative to WENRg+Nic)
WENRg+Nic
WENRg+Nic +A83+SB2
L)
DAPI
Ki67
DAPI
CDX2

Supplemental Figure Legends

Figure S1 – Differentiation of hESCs into endoderm lineages in 2D culture. Related to Figure 1. **A)** Schematic of protocol for *in vitro* differentiation that resulted in foregut and mid/hindgut lineages. **B)** IF staining of stage 3 cells reveals distinct NKX2.1+ domains surrounded by CDX2+ cells. **C)** Phase image of stage 3 cells showing NKX2.1+ domains and surrounding cells. **D)** IF staining of stage 2 cells shows SOX2+FOXA2+ domains and absence of CDX2 expression. **E)** Time-lapse imaging from stage 2 to stage 3 reveals that NKX2.1+domains arise from the SOX2+ domains, whereas CDX2 expression emerges in surrounding cells. **F)** Relative transcript levels of *SOX2* (left) and *CDX2* (right) in cells at each stage of the differentiation protocol; normalized to *GAPDH* expression (values represent linear fold change; n = 2 biological replicates from independent wells of multiple differentiations). All data shown in Figure S1 is for cells derived from H1 hESCs. Scale bars, 500 μm (**B**), 100 μm (**B** inlet), 200 μm (**C**), 150 μm (**D** top), and 300 μm (**D** bottom and **E**).

Figure S2 – Characterization of endoderm-derived tissues made in 3D conditions. Related to Figure 2. **A)** Number of 3D structures produced by input cells (mean ± SEM, n ≥ 3 independent wells of an experiment). **B)** Number of 3D structures produced per well from stage 3 clumps derived from H1 and H9 wt hESCs (mean ± SEM, n = 12 independent wells of an experiment). **C)** Upon separation by micro-dissection of epithelial and mesenchymal components from stage 4 tissues, re-culturing components alone or together shows that epithelium survives and grows in the presence of mesenchyme, but collapses when cultured alone; red arrows point to budding regions in epithelium. **D)** Schematic of FACS-based isolation of ECAD+ and ECAD- cells from endoderm-derived tissues (top), and representative FACS plot of cell separation by ECAD expression (bottom). Scale bar, 300 μm (**C**).

Figure S3 – Characterization and gene expression analysis of spheroids and ECAD+ precursor cells. Related to Figure 3. **A)** Immunohistochemistry of spheroids for lung marker NKX2.1 shows lack of positive staining; adult human lung used as control. **B)** IF staining of spheroids shows co-expression of CDX2 and intestinal crypt marker SOX9. **C)** Number (mean ± SEM, n ≥ 2 independent wells of an experiment) and **D)** diameter (mean ± SEM, n ≥ 18 spheroids spanning 3 independent wells of an experiment) of spheroids produced in MTEC media upon depletion or supplementation of factors. **E)** PCA plot of undifferentiated hESCs and hESC-derived samples shows large variation among phenotypes; microarray data is from GSE89254. **F)** Network diagram of EMAPA showing upregulated (red) and depleted (blue) terms for ECAD+ cells relative to ECAD- cells, and **G)** spheroids relative to ECAD+ cells. Scale bars, 100 μm (**A**) and 50 μm (**B**).

Figure S4 – Generation of hEnS populations in different media conditions. Related to Figure 4. **A)** Whole-well scans; MTEC panel re-used from Figure 2C. **B)** Representative phase image of a spheroid (top) and budding structure (bottom). All images shown in Figure S4 are of hEnS derived from H1 hESCs. Scale bars, 1 mm (**A**) and 300 μm (**B**).

Figure S5 – Characterization of hEnS phenotypes observed in WENRg+Nic and WENRg+Nic+A83+SB2 media. Related to Figure 5. **A)** Comparison of number (mean ± SEM, n = 3 independent experiments) and **B)** diameter of hEnS produced in different media conditions (mean ± SEM, n ≥ 40 spheroids spanning 3 independent experiments, ***p < 0.0001). **C)** Representative IF staining for SOX9 in budding structures in WENRg+Nic. **D)** Proportion of phenotypes identified by AB-PAS staining per tissue section; numbers at the bottom of each bar denote total number of spheres in sections. **E)** Immunohistochemistry of spheroids and budding structures in WENR and WENRg+Nic, respectively, for CDX2 and CK20. **F)** Representative IF staining for P21 and Ki67 of hEnS in various media conditions as well as human small intestine. **G)** Comparison of number (mean ± SEM, n = 3 independent wells of an experiment) and **H)** diameter between hEnS grown in the presence of HLFs and HDFs in WENRg+Nic media (mean ± SEM, n = 37 spheroids spanning 3 independent wells of an experiment). **I)** Representative AB-PAS staining of budding structure

(left) and whole-well scan of population (right) in WENRg+Nic+A83+SB2. **J)** Proportion of structures in WENRg+Nic+A83+SB2 expressing CDX2 or Ki67 per tissue section; numbers at the bottom of each bar denote total number of structures counted. **K)** Relative number of structures produced when hEnS grown in WENRg+Nic are passaged into WENRg+Nic+A83+SB2 media (mean ± SEM, n = 3 independent wells of an experiment). **L)** Representative IF staining for Ki67 (left) and CDX2 (right) in budding structures in WENRg+Nic+A83+SB2. All data shown in Figure S5 is for hEnS derived from H1 hESCs. Scale bars, 100 µm (**C**, **E**, and **L**), 50 µm (**F** and **I** left), and 500 µm (**I** right).

Movie S1 – Self-organization of stage 3 cells into epithelial buds in stage 4 3D Matrigel-based culture conditions. Related to Figure 1. Images were taken every 6 hours for a total of 8 days. Acquired using a Nikon BioStation CT. Scale bar, 100 µm.

Supplemental Experimental Procedures

Maintenance and differentiation of hESCs

H1 and H9 wt hESCs (Wicell Research Institute) were cultured on Matrigel (Corning; #354234) in mouse embryonic fibroblast-conditioned medium (MEF-CM) as previously described (Tomishima, 2008). In preparation for differentiation, hESCs were passaged and seeded in a 48-well format. After seeding, cells were cultured for 2-3 days in MEF-CM, and were then subjected to a 4-stage differentiation protocol.

Differentiation of hESCs into 3D endoderm-derived tissues

In preparation for differentiation, hESCs were passaged and seeded in a 48-well format. After seeding, cells were cultured for 2-3 days in MEF-CM, and were then subjected to a 4-stage differentiation protocol. The basal media used in stage 1 consisted of RPMI 1640 (ThermoFisher; 11875093), 1X non-essential amino acids, 1X GlutaMAX, and 0.05% BSA. In stage 1, hESCs were treated for 3 days with 100ng/ml Activin A (R&D Systems; 338-AC-010), with 25ng/ml WNT3a (R&D Systems; 5036-WN-010), which was supplemented with, 0.2% FBS on day 2, and 2% FBS on day 3. The basal media used in stage 2 and 3 consisted of DMEM/F12 (ThermoFisher; 11320033), 1X non-essential amino acids, 1X GlutaMAX, 0.05% BSA, 0.4µM monothioglycerol (Sigma; M6145), 1X N-2 supplement (ThermoFisher; 17502048), 1X B-27 supplement (ThermoFisher; 17504044), and 50µg/ml L-ascorbic acid. In stage 2, the media was supplemented for 2 days with 200ng/ml Noggin (Peprotech; 120-10C) and 10µM SB431542 (Tocris; 1614). In stage 3, the media was supplemented for 6 days with 100ng/ml WNT3a, 10ng/ml FGF10 (Peprotech; 100-26), 10ng/ml KGF (Peprotech; 100-19B), 10ng/ml BMP4 (Peprotech; 120-05), 20ng/ml EGF (Peprotech; AF-100-15), and 0.05µM all-trans retinoic acid (Sigma; R2625). Cells were washed once with DMEM/F12 between each differentiation stage. Monolayer cultures from stage 3 were collected as aggregates by mechanical scraping followed by gentle trituration. Aggregates were seeded within a 3D matrix of growth factor-reduced Matrigel (GFRM; Corning; #356231; Thick Gel Method, as per manufacturers instructions) diluted in a 1:1 ratio with 100µl MTEC media in a 24-well format. MTEC media (You et al., 2002), comprised DMEM/F12 with HEPES (ThermoFisher; 11330032), 1X non-essential amino acids, 1X GlutaMAX, 1X Insulin-Transferrin-Selenium (ThermoFisher; 51500056), 30µg/ml Bovine Pituitary Extract (ThermoFisher; 13028014), 5% FBS, 25ng/ml EGF, and 10nM all-trans retinoic acid. The 3D matrix was permitted to solidify at 37°C for 30 mins, and then covered with 500µl MTEC media. Cells were cultured at 37°C in a 5% CO_2/air environment, and media was changed every 2 days. Endoderm-derived 3D tissues were typically cultured for 15-20 days.

Generation and culture of hEnS

At the end of stage 4, the gel matrix containing endoderm-derived 3D tissues was dissolved with Cell Recovery Solution (Corning; #354253) for 1 – 1.5 hours on ice. Tissues were washed twice with cold PBS, and dissociated into single cells using TrypLE (ThermoFisher). For ECADHERIN-based FACS, dissociated cells were diluted to 1-5 x 10^6 cells/ml in 1%BSA, 2mM EDTA in PBS, and incubated with mouse anti-ECAD PE (1:100; Santa Cruz; sc-21791 PE) for 1 hour on ice. Sorting was performed on BD FACSAria II, and data was analyzed using FACS DIVA (BD Biosciences). For generation of hEnS, ECAD+ cells were counted (typically 20-30k cells per well), mixed with an equal number of human lung fibroblast (HLF) support cells, and seeded within a 3D matrix (Thick Gel Method) in 75µl MTEC or intestinal media diluted 1:1 with GFRM (total volume 150µl) in a 48-well format. For different intestinal media formulations, the basal media and growth factor concentrations used were exactly as described in other studies (Sato et al., 2009, 2011). After allowing the gel mixture to solidify at 37°C for 30 mins, the gel was covered with 250µl culture media. Cells were cultured at 37°C in a 5% CO_2/air environment, and spheroid formation was observed as early as 5 days post-seeding. With media changes every 4 days, hEnS could be cultured up to 30 days within the same gel matrix without collapsing. For passaging of hEnS, the gel matrix was dissolved with Cell Recovery Solution, then hEnS were dissociated into single cells, counted and seeded as described.

Bacterial and lentiviral infection of hEnS

For infection of hEnS with bacterial cells and LPS, the day before infection, starter cultures of *E. coli* strains Nissile 1917 and O157:H7 were grown overnight in 5ml of LB media at 37°C with shaking. The next morning, bacterial cultures were diluted 1:100 in 3ml of LB and incubated for 3 hours as above to reach their exponential growth phase. The OD_{600} was adjusted to 1x10^8 cfu/ml in antibiotic-free Advanced DMEM/F12 (ThermoFisher; 12634010). In parallel, hEnS were manually plucked out of their gel matrix and also collected in antibiotic-free Advanced DMEM/F12 in culture tubes with the final volume adjusted to about 500ul. hEnS in solution were treated with bacterial cells at an MOI of ~1:50, or 100ng/ml LPS (Sigma, L4391), and incubated for 4 hours at 37°C, 5% CO_2. Supernatant was collected and plated on MacConkey agar to confirm *E. coli* viability. hEnS were washed twice with cold PBS, allowed to settle each time and supernatant aspirated to remove bacteria in solution, then processed for RNA isolation using Trizol LS Reagent (ThermoFisher; 10296010).

For infection of hEnS with H2BGFP-FUCCI virus, hEnS were dissociated into single cells and passaged as described. The day after seeding cells, lentivirus titre was added to the culture media. The media was changed the next day, then 3 days were allowed for transgene integration and hEnS growth, following which 2µg/ml puromycin was supplemented for 10 days, added fresh every 2 days, for selection. For FACS by MKO2-Cdt1 expression, the gel matrix containing spheroids was dissolved, and tissues were dissociated into single cells as described. Cells were diluted in 1%BSA, 2mM EDTA in PBS, and then sorting was performed on BD FACSAria III, with data analyzed using FACS DIVA (BD Biosciences).

Histological staining

Endoderm-derived tissues and hEnS were prepared for histological staining by making formalin-fixed, paraffin-embedded sections. Tissues were harvested from their gel matrix as described, washed twice with PBS, fixed for 1-2 hours at room temperature in 10% neutral-buffered formalin, and then washed again with PBS. Tissues were collected and embedded in HistoGel (ThermoFisher; HG-4000-012) as a plug, then transferred to histology cassettes. Cassettes were taken through an ethanol wash series of increasing concentration and xylene to dehydrate tissue, and then embedded in paraffin at 58°C. 5µm-thick sections were cut using a rotary microtome, floated in a 56°C water bath, mounted onto gelatin-coated histological slides and allowed to dry. H&E, AB/PAS and immunostaining were performed essentially as described in available protocols from R&D Systems for fluorescent and chromogenic staining of paraffin-embedded tissue sections. Antibody usage information is provided below.

RNA extraction and qRT-PCR

RNA from cultured samples was isolated using either Trizol LS Reagent or PicoPure RNA Isolation Kit (ThermoFisher; KIT0204). cDNA was made from total RNA using iScript cDNA Synthesis Kit (Bio-Rad; 1708891). Either SYBR Green-based detection using GoTaq qPCR Master Mix (Promega; A6001) for analysis of *SOX2* and *CDX2* expression, or FAM-based detection using PerfeCTa MultiPlex qPCR SuperMix (Quanta BioSciences; 97065-230) and optimized probes from Universal Probe Library (Roche) for analysis of all other genes, with optimized primer pairs was used for qRT-PCR on a Bio-Rad CFX96. Values were normalized to *GAPDH* using the ΔCt method and to calculate fold-change relative to reference samples. Primer sequences are provided below. For generation of the clustergram, z-scores calculated from ΔCt values were used to create a heatmap in NetWalker, and non-supervised hierarchical clustering of heatmap samples was done using SABiosciences qPCR Array data analysis web portal: http://pcrdataanalysis.sabiosciences.com/pcr/arrayanalysis.php

Microarray and PCA

Microarray data was processed from CEL files using the *oligo* package from Bioconductor. Samples were batch corrected using the *limma* package method *removeBatchEffect*. Differential gene expression was calculated using linear models by the *limma* package. Ranked gene expression data was assessed by Gene Set Enrichment Analysis to identify enriched developmental and biological processes in the different samples. A custom gene set was developed using the EMAPA ontology to assess enrichment of gene sets annotated to different anatomical structures, combined with markers identifying specific intestinal lineages obtained from a dataset profiling single intestinal cell types (GSE62270). GSEA data files were processed in Cytoscape using the Enrichment Map plugin to develop network graphs of ontology term enrichments.

To compare our enterospheres (GSE89254) with other human major organ systems, we used two different array data sets GSE2361 and GSE30803. To merge the data sets collected on different platforms, we mapped all probe IDs to gene symbols and merged redundant symbols by their mean signal. Data sets were then joined by gene symbol on rows and samples on columns. Data were all log2 transformed, normalized and batch corrected. To compare our enterospheres with other hPSC-derived intestinal organoids, we used data sets obtained from (https://github.com/hilldr/Finkbeiner_StemCellReports2015) and GSE56930. RNA-seq data was transformed into log2 counts per million expression values. Data sets were then merged similar to above on gene symbols. Data was normalized and batch corrected.

Heat maps and PCA and correlation plots were all generated from the top 10th centile of genes (1684 genes) filtered with a standard deviation filter. These highly variable genes should be enriched in information and enable separation of different cell types with out using a biased selection system. Heat maps were generated using the R package *pheatmap* using Euclidian distance metrics and complete clustering. PCA was performed and graphed using the R functions *prcomp* and *plot*. Correlation plots were generated using the R package *corrplot* with hierarchical clustering.

Image processing, data analysis and statistics

ImageJ was used for counting spheroids, diameter measurements and processing of histology staining montages. Whole-well phase images of spheroids were stitched together from individual fields using Grid/Collection Stitching plugin on ImageJ (Preibisch et al., 2009). Automated image analysis was performed using the CellProfiler software package. Graphs from quantitative data were created on GraphPad Prism 5, and significant differences in sample means were assessed using two-tailed unpaired t-test.

Antibody usage for IHC-P/IF and ICC/IF

**Antibody/protein**	**Usage**	**Vendor**	**Catalog no.**
	IHC-P/IF		
ECAD	1:100	Santa Cruz	sc-21791

NCAD	1:500	Abcam	ab18203
αSMA	1:200	Abcam	ab5694
SHH	1:100	Santa Cruz	sc-1194
P63	1:100	Santa Cruz	sc-8431
NKX2.1	1:100	Santa Cruz	sc-13040
SOX2	1:400	BD	#561469
CDX2	1:800	Biogenex	CDX2-88
SOX9	1:500	Abcam	ab76997
VIL1	1:100	Santa Cruz	sc-7672
SI	1:100	Santa Cruz	sc-27603
MUC2	1:100	Santa Cruz	sc-15334
LYZ	1:100	Santa Cruz	sc-27956
P21	1:400	Cell Signaling	#2947
Non-phospho β-catenin	1:400	Cell Signaling	#8814
Ki67	1:400	Cell Signaling	#9449
ICC/IF			
SOX2	1:400	BD	#561469
FOXA2	1:200	Santa Cruz	sc-9187
NKX2.1	1:200	Santa Cruz	sc-13040
CDX2	1:1000	Biogenex	CDX2-88

Chromogenic IHC-P was performed by technical staff at the HRLMP in St. Joseph's Hospital (Hamilton, Ontario, Canada). Antibody information:

NKX2.1/TTF-1: Clone – 8G7G3/1; Isotype – IgG1, kappa
CDX2: Clone – DAK-CDX2; Isotype – IgG1, kappa
CK20: Clone – Ks20.8; Isotype – IgG2a, kappa

QRT-PCR primer sequences

**Primer name / gene**		**Sequence (5' -> 3')**
For use with GoTaq qPCR Master Mix		
SOX2-F		TACAGCATGTCCTACTCGCAG
SOX2-R		GAGGAAGAGGTAACCACAGGG
CDX2-F		TCCGTGTACACCACTCGATATT
CDX2-R		GGAACCTGTGCGAGTGGAT
GAPDH-F		GGTATCGTGGAAGGACTCATGAC
GAPDH-R		ATGCCAGTGAGCTTCCCGTTCAG
For use with PerfeCTa MultiPlex qPCR SuperMix and probes from UPL		
**Primer name / gene**	**Probe #**	**Sequence (5' -> 3')**
LGR5-F	78	accagactatgcctttggaaac
LGR5-R		ttcccagggagtggattctat
OLFM4-F	24	atcaaaacacccctgtcgtc
OLFM4-R		gctgatgttcaccacaccac
ASCL2-F	55	gcaccaacacttggagatttt
ASCL2-R		aatggattctctgtgcccttag
VIL1-F	87	ttgccacaattccctgagat
VIL1-R		cttggtcatggtgagtgagc
LYZ-F	68	ccgctactggtgtaatgatgg
LYZ-R		catcagcgatgttatcttgcag
MUC2-F	1	gctgctatgtcgaggacacc
MUC2-R		gggaggagttggtacacacg
GAPDH-F	60	agccacatcgctcagacac

GAPDH-R		gcccaatacgaccaaatcc
TNFα-F	29	cagcctcttctccttcctgat
TNFα-R		gccagagggctgattagaga
IL6-F	40	gatgagtacaaaagtcctgatcca
IL6-R		ctgcagccactggttctgt
IL8-F	72	gagcactccataaggcacaaa
IL8-R		atggttccttccggtggt
DEFB4A-F	35	tcagccatgagggtcttgta
DEFB4A-R		ggatcgcctataccaccaaa

Supplemental References

Preibisch, S., Saalfeld, S., and Tomancak, P. (2009). Globally optimal stitching of tiled 3D microscopic image acquisitions. Bioinformatics *25*, 1463–1465.

Sato, T., Vries, R.G., Snippert, H.J., van de Wetering, M., Barker, N., Stange, D.E., van Es, J.H., Abo, A., Kujala, P., Peters, P.J., et al. (2009). Single Lgr5 stem cells build crypt-villus structures in vitro without a mesenchymal niche. Nature *459*, 262–265.

Sato, T., Stange, D.E., Ferrante, M., Vries, R.G.J., Van Es, J.H., Van den Brink, S., Van Houdt, W.J., Pronk, A., Van Gorp, J., Siersema, P.D., et al. (2011). Long-term expansion of epithelial organoids from human colon, adenoma, adenocarcinoma, and Barrett's epithelium. Gastroenterology *141*, 1762–1772.

Tomishima, M. (2008). Conditioning pluripotent stem cell media with mouse embryonic fibroblasts (MEF-CM). In StemBook, (Cambridge (MA): Harvard Stem Cell Institute),.

You, Y., Richer, E.J., Huang, T., and Brody, S.L. (2002). Growth and differentiation of mouse tracheal epithelial cells: selection of a proliferative population. Am. J. Physiol. Lung Cell. Mol. Physiol. *283*, L1315–L1321.
