# Supplementary figures and images for: Functional Enterospheres Derived In Vitro from Human Pluripotent Stem Cells
Source: Stem Cell Reports. 2017 Aug 31;9(3):897–912. doi: 10.1016/j.stemcr.2017.07.024 (PMC5599260; doi:10.1016/j.stemcr.2017.07.024)

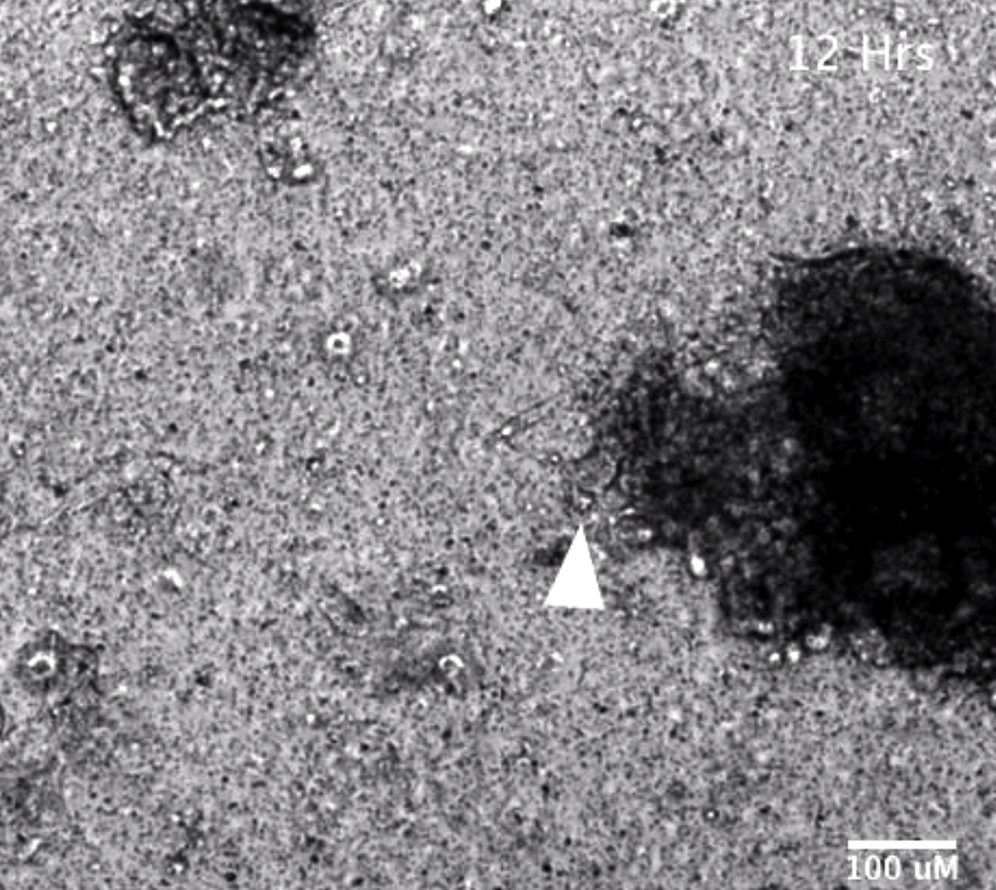

Supplement: Movie S1. Self-Organization of Stage 3 Cells into Epithelial Buds in Stage 4 3D Matrigel-Based Culture Conditions, Related to Figure 1 — Images were taken every 6 hr for a total of 8 days. Acquired using a Nikon BioStation CT. Scale bar, 100 μm. [file mmc2.jpg]
